# A Mechanistic Model of Early FcεRI Signaling: Lipid Rafts and the Question of Protection from Dephosphorylation

**DOI:** 10.1371/journal.pone.0051669

**Published:** 2012-12-17

**Authors:** Dipak Barua, Byron Goldstein

**Affiliations:** 1 Theoretical Biology and Biophysics Group, Theoretical Division, Los Alamos National Laboratory, Los Alamos, New Mexico, United States of America; 2 Center for Nonlinear Studies, Los Alamos National Laboratory, Los Alamos, New Mexico, United States of America; Cornell University, United States of America

## Abstract

We present a model of the early events in mast cell signaling mediated by FcεRI where the plasma membrane is composed of many small ordered lipid domains (rafts), surrounded by a non-order region of lipids consisting of the remaining plasma membrane. The model treats the rafts as transient structures that constantly form and breakup, but that maintain a fixed average number per cell. The rafts have a high propensity for harboring Lyn kinase, aggregated, but not unaggregated receptors, and the linker for the activation of T cells (LAT). Phosphatase activity in the rafts is substantially reduced compared to the nonraft region. We use the model to analyze published experiments on the rat basophilic leukemia (RBL)-2H3 cell line that seem to contradict the notion that rafts offer protection. In these experiments IgE was cross-linked with a multivalent antigen and then excess monovalent hapten was added to break-up cross-links. The dephosphorylation of the unaggregated receptor (nonraft associated) and of LAT (raft associated) were then monitored in time and found to decay at similar rates, leading to the conclusion that rafts offer no protection from dephosphorylation. In the model, because the rafts are transient, a protein that is protected while in a raft will be subject to dephosphorylation when the raft breaks up and the protein finds itself in the nonraft region of the membrane. We show that the model is consistent with the receptor and LAT dephosphorylation experiments while still allowing rafts to enhance signaling by providing substantial protection from phosphatases.

## Introduction

Lipid rafts are ordered regions of the plasma membrane enriched in cholesterol and glycosphingolipids [1]–[4] that are thought to play important roles in immune recognition receptor-mediated signaling [5]–[7]. These regions of the membrane possess distinct structural and compositional properties that allow them to harbor some signaling proteins [8]–[14] and exclude others [5], [15], [16]. The spatial partitioning of signaling proteins into the raft and nonraft part of the membrane presumably determine the interactions of subsets of signaling proteins [16]. Significant efforts have been invested in understanding the consequences of the partitioning of proteins in rafts on signaling mediated by various immunoreceptors [16]–[19]. These efforts have led to “the lipid raft hypothesis” which proposes that lateral heterogeneities in the membrane are intimately involved in a variety of cellular processes including cell signaling [20].

Lipid rafts have been extensively studied in the context of signaling mediated by FcεRI, the high-affinity receptor for IgE, in rat basophilic leukemia (RBL) cells. These studies have revealed details as to how the different proteins in this signaling pathway are partitioned by lipid rafts in the cell membrane [21]–[26]. Receptors (FcεRI) are partitioned in a stimulation-dependent manner [21], [27], [28]. In the absence of stimulation, receptors primarily exist as monomers, which indifferently distribute into the raft and nonraft parts of the plasma membrane. Upon stimulation by a polyvalent ligand, receptors form dimers and larger aggregates, which partition predominantly into the lipid rafts. Beside the receptors, other primary membrane proteins that partition into rafts and that are involved in FcεRI signaling are the Src-family protein tyrosine kinase (PTK) Lyn, which initiates receptor phosphorylation, and the scaffold protein linker for the activation of T cells (LAT) [9], [22], [25], [29]–[31]. Lyn is posttranslationally modified with a palmitate and a myristate chain, and LAT is modified with dual palmitate chains. The palmitate chains allow both these proteins to partition into the rafts with high propensities [9], [29], [30], [32]. Ligand-induced aggregation, which causes more receptors to move into the rafts, is expected to enhance the co-localization of receptors with Lyn and LAT and promote their interactions [19]. From immunogold labeling of membrane sheets it was observed that upon FcεRI cross-linking, large rafts containing LAT formed that often intersected osmiophilic patches containing the receptor [33]. In resting T cells LAT and the T cell receptor were in separate membrane domains, but after T cell activation these domains concatenated, juxtaposing TCRs and LAT along the domain borders [34]. In RBL cells LAT is phosphorylated by Syk that is bound to FcεRI [35]. Syk is recruited from the cytosol to the FcεRI γ-chain after receptors are cross-linked by ligand and phosphorylated by Lyn [36], [37]. We assume that regions containing both receptors and LAT must be present on the cell surface for receptor associated Syk to phosphorylate LAT. When we present our model of FcεRI cell signaling, we consider a simplified picture of a cell surface and take the surface to be composed of a set of rafts of identical composition with the remainder of the surface composed of a homogeneous nonraft region.

Rafts make up a large percentage of the lipid component of the plasma membrane [19], [38], [39]. The enhancement of the concentration of raft-associated proteins compared to if these proteins were uniformly distributed over the plasma membrane is no more than a factor of two or three. Such enhancement in concentrations undoubtedly influences signaling, but more as a perturbation than a robust mechanism. A body of evidence suggests that it is the proteins that are excluded from the rafts that may be key in regulating signaling. Baird, Holowka, and colleagues have proposed that the exclusion of transmembrane protein tyrosine phosphatases (PTPases) from lipid rafts may be a general mechanism for the regulation of immunoreceptor signaling [19], [24], [25]. Being excluded by the lipid rafts, transmembrane PTPases cannot readily access the raft-localized proteins, thereby helping to sustain protein phosphorylation and signaling. They showed that the co-expression of PTPα, a transmembrane PTPase, with FcεRI and Lyn, suppressed spontaneous but not antigen-dependent FcεRI phosphorylation by Lyn in CHO cells [25]. In contrast, a chimeric PTPα capable of accessing lipid raft domains failed to reconstitute the antigen-dependent FcεRI phoshporylation in their experiment [25].

**Figure 1:**
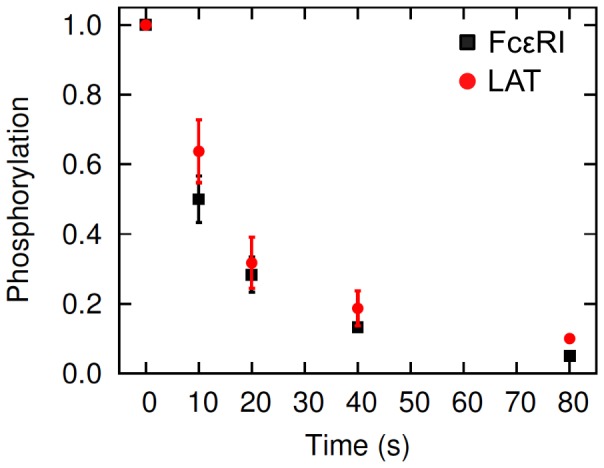
FcεRI and LAT dephosphorylation in the hapten-inhibition experiment of Peirce and Metzger [40]. The figure is modified from Fig. 4B in Peirce and Metzger [40]. Square symbols represent FcεRI and the circles represent LAT. The experiment is detailed in Peirce and Metzger [40] and in Materials and Methods of this study. RBL cells were first stimulated with a polyvalent antigen to induce receptor crosslinking and signaling (before time zero in the figure), and then excess monovalent hapten was added (at time zero) to break up the receptor crosslinking. Subsequently, amounts of phosphorylated FcεRI and LAT were assayed at different time points. The figure shows the fraction of phosphorylated FcεRI and LAT remaining after hapten addition.

The proposed mechanism of raft-mediated exclusion of PTPases in RBL signaling has been challenged. Peirce and Metzger [40] have reported that proteins can dephosphorylate at similar rates in raft and nonraft parts of the plasma membrane. They studied the kinetics of dephosphorylation (by endogenous PTPases) of LAT and unaggregated FcεRI in RBL cells, respectively a raft- and a nonraft-associated protein [40]. They first stimulated receptor crosslinking in cells with a multivalent antigen, and then added a high affinity, monovalent hapten, in large excess to displace the antigen from the receptors and breakup receptor aggregates. The hapten caused rapid dissociation of crosslinked receptors and presumably, relocation of the resulting receptor monomers to regions outside the lipid rafts. The consequent disruption of signaling led to spontaneous dephosphorylation of proteins that were already phosphorylated prior to hapten addition. They observed that LAT and FcεRI dephosphorylated at approximately equal rates, despite presumably being localized in raft and nonraft compartment of the membrane (results from their experiment have been reproduced in Figure 1; also see figure 4B in [40]). From the results, they concluded that the raft-exclusion of PTPases did not contribute to protection of proteins from dephosphorylation.

The apparently contradictory findings from the Baird-Holowka and Metzger laboratories motivated us to extend a previous model of cell signaling to include lipid rafts in order to investigate the potential roles of lipid rafts in early events of FcεRI mediated signaling. In this study, we develop and analyze a model of FcεRI signaling, considering partitioning of signaling proteins into raft and nonraft domains in the cell membrane. Earlier Faeder et al. [41] published a mechanistic model of membrane-proximal FcεRI signaling, where the membrane was taken to be homogenous with the proteins distributed randomly. We extend this model to include lipid rafts, and introduce into the model LAT and the adaptor protein Grb2 that binds to phosphorylated LAT.

Analysis of the model reveals that there may be no contradiction between experiments of the Baird-Holowka lab [24], [25] and the Metzger lab [40]. Lipid rafts can contribute significantly to enhancing protein phosphorylation through PTPase exclusion, and yet be consistent with the dephosphorylation experiments of Peirce and Metzger [40]. Using the model, we simulate the dephosphorylation experiments from Metzger’s laboratory [40], [42], and analyze the kinetics of LAT and FcεRI dephosphorylation. Our analyses reveal that, within a reasonable parameter regime, LAT and FcεRI can dephosphorylate at similar rates, as observed [40], despite strong lipid raft-mediated protection of LAT from membrane PTPase activities. In the model, the apparent contradiction is resolved as a result of postulating that lipid rafts have finite lifetimes, on the order of seconds. Therefore, even assuming absolute protection where lipid rafts are completely inaccessible to PTPases, proteins will still be subject to dephosphorylation due to the transient nature of raft partitioning of proteins as a result of the raft’s lifetime. We expect that absolute protection is unlikely, because not only can membrane PTPases access the rafts with variable capabilities, but also the cytosolic PTPases, whose activities presumably are not affected by the rafts, can participate in dephosphorylatoin of the raft-localized proteins. Mao and Metzger have reported that in RBL cells some soluble PTPases contribute to the dephosphorylation of FcεRI and Lyn [42]. Here we show that in this later case, where raft protection is not absolute, the finite lifetime of lipid rafts together with the partial raft-seclusion of proteins and PTPases can give rise to similar overall dephosphorylation rates for the raft and nonraft-associated proteins.

## Materials and Methods

### FcεRI Signaling Model

We extend the IgE receptor-signaling model of Faeder et al. [41] to include lipid rafts and the possibility that they exclude PTPases. Additionally, we introduce two proteins that were not in the original model, LAT and Grb2, albeit in a simplified form. LAT is a lipid raft-associated scaffolding protein [9], [30], which in mast cells is phosphorylated by Syk on multiple tyrosine sites [43]–[45]. The three terminal tyrosines of LAT, when phosphorylated, bind to the SH2 domain of Grb2 [45]. In the model, the simplified LAT contains a single tyrosine site, which is phosphorylated by activated Syk. As for Grb2, we ignore complexes in the cytosol that contain two Grb2 molecules, and we allow only single Grb2 binding with LAT. Grb2 is included in the model to incorporate the protective effects on LAT phosphotyrosines by Grb2 or other proteins that bind through their SH2 domains to phosphotyrosines on LAT. For the dephosphorylation experiment we analyze, we expect the simplifications we have introduced to be unimportant as the dephosphorylation curve for LAT (Figure 1) is primarily a result of the dissociation of the SH2 domain of Grb2 and the rate of dephosphorylation of the phosphotyrosines on LAT.

**Figure 2:**
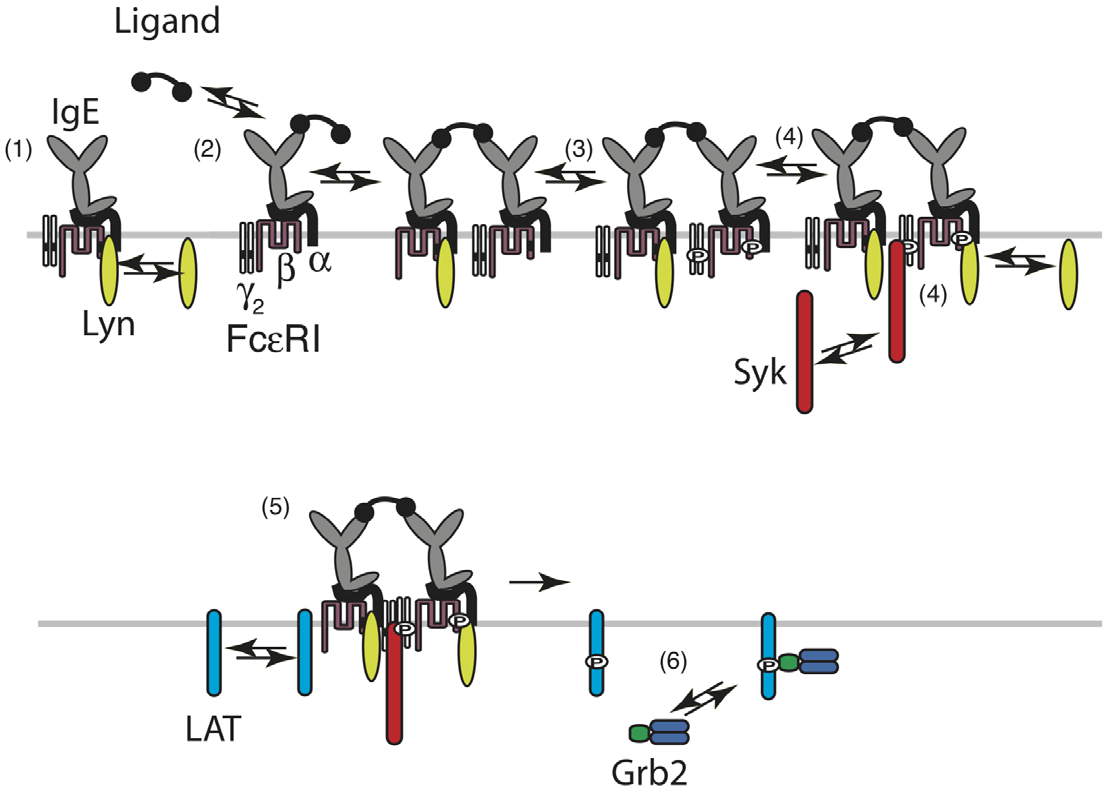
Binding and phosphorylation reactions in the model that occur both in raft and in nonraft compartments. 1) Constitutive association of Lyn unique domain with the β subunit of the receptor. 2) Binding and cross-linking of IgE-FcεRI complexes by a multivalent ligand; 3) Transphosphorylation of the β and γ ITAMs by constitutively associated Lyn. 4) Recruitment of Lyn through its SH2 domain to the phosphorlated β ITAM and recruitment of Syk through its two SH2 domains to the doubly phosphorylated γ ITAM. 5) Michaelis-Menton interaction of a receptor aggregate containing Syk with LAT resulting in a phosphorylated LAT. 6) Binding of Grb2 to phosphorylated. Not shown are the reactions that dephosphorylate unprotected tyrosines.

The original model was formulated using a detailed approach that incorporated the domain and motif interactions of key signaling proteins governing membrane proximal events in the signaling pathway. The proteins included were the FcεRI receptor complex and its extracellular ligand (covalently conjugated IgE dimer), membrane-associated Src-family tyrosine kinase Lyn, and cytosolic tyrosine kinase Syk. The essential structure-function of these proteins and their implications in the pathway have been detailed by Faeder et al. [41]. The basic interactions are outlined in Figure 2.

### Lipid Raft Model of the Plasma Membrane

In our model, the plasma membrane is divided into raft and nonraft compartments. Separately, proteins within the compartments are well mixed. The raft compartment is the sum of all lipid rafts in the membrane, whereas the nonraft compartment represents all the membrane outside the raft domains. What is important in the model is the total surface area covered by rafts, the surface area covered by a single raft, and the number of Lyn, LAT and receptors that are present in the raft. For simplicity, we assume all rafts are of the same dimension, covering an area equal to a disk of radius 100 nm. We take the cell surface area for a rat basophilic leukemia (RBL) cell to be 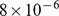 cm^2^ and assume the raft compartment occupies ∼30% of the plasma membrane [46]. Thus, the number of rafts in our model is about 8,000 per cell. We further assume that all lipid rafts randomly appear and disappear in the plasma membrane with the same lifetime, and that their rate of appearance and disappearance is such that the density of lipid rafts remains constant. We initially take the lifetime 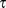 of the rafts to be 10 s, i.e., on average, a raft forms and turns over at a rate of 0.1 s^−1^, but we will see that our results are relatively insensitive to the raft lifetime.

### Lipid Raft Partitioning of Signaling Proteins

Membrane proteins in our model are allowed to segregate between raft and nonraft compartments based on their propensities for raft/nonraft partitioning. We assume that unaggregated FcεRI does not favor the raft environment so that in the absence of aggregation the concentration of receptor is the same in and out of lipid rafts. We therefore take 30% of monomeric FcεRI to partition into the raft compartment (partition coefficient 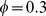) because the raft compartment constitutes 30% of the cell membrane in the model. For a ligand cross-linked receptor dimer, which predominantly partitions into lipid rafts [21], [27], [28], we take 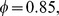 which we obtained by extrapolating experimental data in Kovarova et al. [29] that reported a variable, concentration-dependent efficiency of solubilizing detergent Triton X-100 in determining raft partitioning of aggregated FcεRI. Using 0.06 or 0.1% Triton X-100 to solubilize RBL cells, the authors identified 65.6 and 52.9% raft-association of aggregated FcεRI, respectively. By extrapolating these data to 0% Triton X-100, we obtained an estimated 85% raft partitioning of aggregated receptors, which we used in the model. For Lyn, which also predominantly partitions into lipid rafts [16], [25], [29], we also take 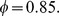 Variable extents of Lyn partitioning have been reported in the literature [22], [25], [29]; Field et al. [22] reported that at least 30% of Lyn partitions into lipid rafts, although later studies identified higher extents of partitioning: approximately 55% has been reported in Kovarova et al. [29], and more recently >60% has been reported in Young et al. [25]. It important to note that, in our model, Lyn represents only the active fraction of cellular Lyn [41], not the entire population of Lyn. For LAT, we take 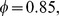 the same as the aggregated FcεRI and Lyn. There is conflicting evidence as to whether aggregated receptors and LAT are found in the same microdomains on the RBL cell surface. Zhang et al. [9] identified LAT as a raft-localized protein and they showed that palmitoylation of LAT plays an essential role in its raft partitioning. Experiments involving detergent extraction have also identified LAT as being a raft-localized protein [9], [30], [47]. Visualizing immunogold labeled RBL cell membranes using transmission electron microscopy, Wilson et al. [33], [48] found that aggregated receptor and LAT partitioned into different surface domains. However, Das et al. [49] using real-time cross-correlation image analysis found that after RBL cell stimulation, LAT and Lyn co-localized with the receptor. In the dephosphorylation experiments of Peirce and Metzger [40], as soon as receptor-aggregates dissociated, both the receptor and LAT immediately commenced to dephosphorylate, indicating that the receptor and LAT were in intimate communication. In our model we assume that Lyn and LAT are found in the same rafts and the aggregated receptors partition into these rafts.

Because in the model the concentration of rafts does not change, although individual rafts constantly form and dissolve with a lifetime of 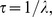 the fraction of a protein in the raft compartment at equilibrium, 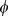 is equal to the fraction of time the protein spends in the raft compartment, i.e.,
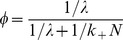
(1)Here, 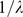 is the average lifetime of a lipid raft, 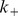 is the two dimensional forward rate constant of the protein in the nonraft region of the membrane to enter the raft, and 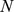 is the density of lipid rafts in the cell membrane. The lumped parameter 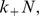 obtained by multiplying the two-dimensional forward rate constant with the raft density, represents the transition rate of a protein from nonraft region to raft region of the membrane (and therefore its inverse is the average time a protein spends in diffusing through the nonraft region between successive captures by the raft domains). As mentioned before, the lifetime of lipid rafts 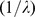 is initially assumed to be 10 s in our model, and additionally, the partition coefficients 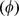 for different proteins are based on their known propensity for raft partitioning (Table S1). Therefore, by introducing these quantities in the above equation, we can estimate the unknown raft-association parameter 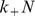 for individual proteins in our model. For example, for 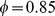 and 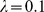 s^−1^, 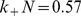 s^−1^, and in one cycle LAT would spend 10 s in the lipid raft and 1.8 s outside it. Whatever the lifetime of the raft is, for a given partition coefficient the fraction of the time a protein spends in the raft is the same.

Based on the partition coefficients for individual protein molecules discussed above, we set the criterion for the partitioning of protein complexes. When Lyn is bound to a receptor that is not in an aggregate we assume the bound complex partitions in the same way as Lyn does, i.e., the palmitoylation of Lyn determines the partitioning of the complex. Further, we assume that the interactions with cytosolic proteins do not alter the partitioning of the membrane proteins.

### Protein Dephosphorylation in Raft and Nonraft Compartments

Signaling proteins can be subject to differential exposure to PTPases in the raft and nonraft regions due to the partitioning of PTPases. Many membrane PTPases presumably are raft-excluded, because they do not contain the fatty acyl chains, such as palmitate, which targets proteins into the lipid rafts [29]. Moreover, many membrane PTPases are transmembrane proteins [50], and transmembrane proteins usually associate with lipid rafts poorly [51]. For example, the transmembrane PTPase CD45 in Jurkat cell has been identified as a raft-excluded protein [52]. Another transmembrane PTPase, PTPα, is also excluded in the nonraft region of the membrane [25].

In the model, we quantify the effects of lipid raft exclusion of PTPases on protein phosphorylation through the parameter 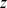 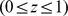, which represents the ratio of the dephosphorylation rate of a protein inside the lipid rafts to that in the nonraft region. We assume that each protein dephosphorylates at an intrinsic rate in the nonraft compartment, and we scale this rate by factor 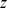 when the protein is inside the raft compartment.

In the model, 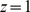 makes the raft compartment indistinguishable from the nonraft compartment in terms of the PTPase activities, i.e. when 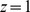 raft domains provide no protection for phosphorylated proteins from dephosphorylation. On the other hand, when 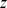 is 0, there is no PTPase activity within the raft compartment, and therefore rafts provide absolute protection to proteins from dephosphorylation. An intermediate value for 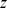 indicates that lipid rafts are partially accessible to PTPases and hence proteins are partially protected from dephosphorylation, with the level of protection depending on the value of 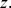


In the model, phosphorylation of FcεRI β and γ ITAMs, Syk, and LAT is determined by 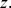 The sites of phosphorylation of Lyn are not considered. We take Lyn to have a five-fold reduced kinase activity in the nonraft compartment compared to the raft compartment, as observed [24]. This difference in kinase activity of Lyn in the two compartments, arising from the increased phosphorylation of its activation loop tyrosine in lipid rafts [24], takes into account the effects of membrane separation of the PTPases.

### FcεRI and LAT Dephosphorylation/Hapten Inhibition Model

Metzger and colleagues used monovalent hapten to disrupt IgE-induced receptor crosslinking and measured the time course of FcεRI and LAT dephosphorylation after hapten addition [40], [42]. We simulated their experiments by introducing monovalent hapten as an additional component for receptor binding in our FcεRI model. As described before, we extended the original FcεRI signaling model of Faeder et al. [41] by including LAT and Grb2. This extension was necessary to simulate the LAT dephosphorylation experiment. Phosphorylated LAT serves as a scaffold for various cytosolic proteins carrying SH2 domains. Grb2 is one of these LAT binding proteins that associates with LAT through its single SH2 domain. Grb2 was introduced in the model to reflect its protective effects on LAT phosphotyrosines. Previously Faeder et al. [41] had shown that in RBL cells the binding of Lyn and Syk SH2 domains protects the phosphorylated tyrosines of FcεRI β and γ ITAM, respectively, from PTPase-mediated dephosphorylation. They estimated that unbound phosphotyrosines on the FcεRI β and γ ITAMs dephosphorylate with a rate constant 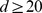 s^−1^, i.e., the mean lifetime of a naked ITAM phosphotyrosine is 0.05 s or less.

In the model we simulate a dephosphorylation experiment by first stimulating signaling with a bivalent ligand that binds and crosslinks reversibly IgE-FcεRI complexes on RBL cell. What makes the cell an RBL cell in our model is that the concentrations used for FcεRI, Lyn, Syk, and LAT are those determined for RBL-2H3 cell. The bivalent ligand was added in the simulation at 1 nM concentration (close to the optimal concentration ∼5 nM) in order to induce substantial receptor crosslinking/signaling in the model. The model generated a network of 348 protein species and 3,447 unidirectional reactions after stimulation (formulation and structure of such models are detailed in Faeder et al. [41]). We allowed the simulation to proceed to steady state, and collected the concentrations of all of the 348 model-generated protein species. Subsequently we used these steady-state concentrations as the initial condition in the hapten inhibition experiment. Monovalent hapten with the rate constants for DNP-εNH_2_-caproate (DCT), the hapten used in the experiments [40], was added in excess (∼100 µM) to disrupt the reversible ligand-induced receptor crosslinking in the model. Hapten was allowed to bind, and occupy essentially all receptors by replacing the bivalent ligand. Dephosphorylation of proteins (FcεRI ITAMs, Syk and LAT) was allowed to proceed spontaneously after the hapten-induced disruption of the receptor crosslinking.

### Diffusion-limited Two-dimensional Forward Rate Constant (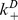)

In our model we must make sure that the rate constant for a protein outside a raft to enter a raft, 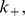 as predicted from Eq (1), is always less than the diffusion limited value, 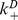 The diffusion-limited two-dimensional forward rate constant for a surface protein interacting with a raft represents the permissible highest rate at which a protein can be captured by lipid rafts in the plasma membrane in the absence of any convective process. The parameter 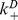 can be derived analytically for targets (rafts) that have only finite lifetimes, as shown previously by Goldstein et al. [53] for an analogous system. We use the formulation of Goldstein et al. [53] to calculate the 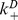 as follows:
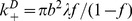
(2)where,



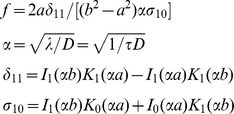



In the above formulation, 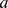 is the radius of lipid rafts, and 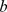 is the distance from the center of a lipid raft to the boundary of the annular nonraft region associated with each raft 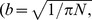 where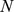 is the density of rafts in the plasma membrane). The only place in the model where we need to assume that the raft is circular is in calculating 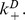 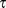 is the average lifetime of lipid rafts. 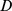 is the sum of the diffusion coefficient of the diffusing protein in the nonraft compartment and the diffusion coefficient of the raft. 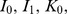 and 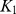 are the modified Bessel functions of the first and second kind.

We assume that the lipid raft diffuses much faster than a protein in the plasma membrane outside the raft. Pralle et al. [54] determined the raft diffusion coefficient, 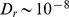 cm^2^/s, while protein diffusion coefficients are typically in the range 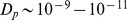 cm^2^/s [55], [56]. We therefore replace_
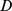
_ for individual proteins in the above expression with 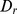. With this replacement, Eq (2) calculates a single diffusion limited forward rate constant 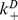 for all the membrane proteins in the system.

### Simulation of Experiment for Lyn Palmitoylation Site Mutation

We simulated the experiment of Kovarova et al. [29] that investigated the effects of mutating Lyn at its palmitoylation site on phosphorylation of FcεRI, Syk, and LAT. In our model, what makes it a mutated Lyn is its weaker ability to partition into lipid rafts as compared to that of a wild type Lyn. In their study, Kovarova et al. [29] expressed the wild type and mutated Lyn in RBL cells, and quantified their raft partitioning through immunoblotting after detergent (Triton X-100) solubilization of the cells. From their data (Table 1 in [29]), we obtained partition coefficients 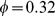 and 0.06 for the wild type and mutated Lyn, respectively. For the wild type Lyn, we carried out simulations using 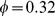 (instead of 0.85, the default value used in other calculations). However, for the mutated Lyn, in addition to using 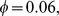 we now assume that a mutated Lyn associated with a nonaggregated receptor does not influence the partitioning of the receptor. The mutated Lyn-receptor complex is equally likely to be anywhere in the cell membrane 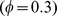.

### Model Implementation

The model is an ODE-based kinetic model simulated as in Faeder et al. [41]. The model is coded and implemented using the rule-based modeling software BioNetGen [57]. The model input file (BioNetGen code) is included in the Text S1.

## Results

### Lipid Raft Exclusion of Membrane PTPases and Effects on Protein Phosphorylation

We consider an RBL cell sensitized with 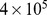 monoclonal IgE and look at the predicted dose-response curve of the cells to a range of concentrations of a bivalent ligand that reversibly binds and crosslinks the surface IgE-FcεRI complexes.

**Figure 3:**
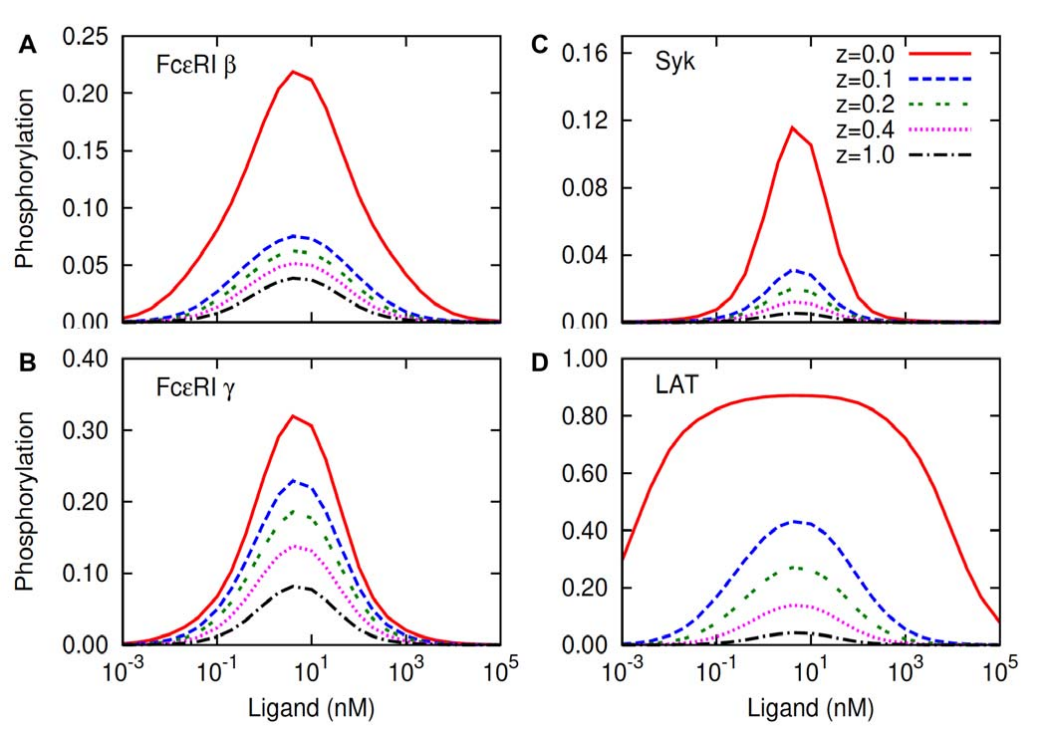
Predicted effects of lipid raft protection on protein phosphorylation. Ligand dose-dependent phosphorylation of (A) FcεRI β, (B) FcεRI γ, (C) Syk, and (D) LAT in the model is shown for different levels of lipid raft protection. Phosphorylation is represented as the fraction of each protein in the phosphorylated state. Parameter 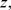 which represents the level of raft protection (Materials and Methods), has the following interpretations: 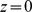 represents absolute protection (proteins do not dephosphorylate inside lipid rafts); 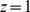 represents no protection (proteins dephosphorylate at equal rates inside and outside lipid rafts); and 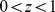 represents partial protections (proteins dephosphorylate at slower rates inside lipid rafts than outside lipid rafts). In simulations, default values (Table S1) are used for parameters not shown in the figure.

The steady-state protein phosphorylation in the model is shown in Figure 3 as a function of the ligand concentration at various levels of lipid raft protections. (Because internalization of receptors and down regulation of signaling molecules is not in the model, at long times phosphorylation goes to an elevated steady state value rather than returning to a basal level.) The parameter 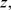 that accounts for a lipid raft’s ability to exclude the PTPases, represents the level of protection of phosphorylated proteins inside the lipid rafts. The parameter is the ratio of the rate at which a protein dephosphorylates inside the raft compartment to the rate at which it dephosphorylates outside the raft compartment (Materials and Methods). The value of 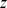 is between zero and one, where zero represents absolute protection by the rafts and one represents no protection by the rafts. For 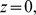 proteins inside the rafts do not dephosphorylate. For 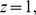 proteins dephosphorylate inside and outside rafts at equal rates. The results in Figure 3 show pronounced effects of lipid raft-mediated protection on protein phosphorylation. Relative to the base case, 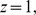 phosphorylation when protection is absolute 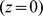 is increased by approximately 6, 4, 21, and 20 fold for FcεRI β, γ, Syk, and LAT, respectively, near the optimal ligand concentration for IgE aggregate formation maximal (∼5 nM) (Figure 3). The effect is most pronounced for LAT where the predicted phosphorylation remains over a broad range of ligand concentrations extending as low as 10^−2^ nM (Figure 3D). Such stimulation for bivalent ligand concentrations 100-times lower than their 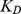 (in the simulation, 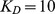 nM for the bivalent ligand) are not seen in experiments. The predictions in Figure 3D suggest that 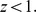 Cytosolic PTPases as well as membrane associated PTPases that may act at the perimeter of the rafts, as suggested by Peirce and Metzger [40], would all contribute to protection being less than absolute. We therefore expect that at best, lipid rafts provide only partial protection 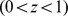 to the phosphorylated proteins. However, even in the regime of partial protection, the effect can be considerable. As evident in Figure 3, allowing a modest access of PTPases into the raft compartment 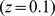 drastically reduces protein phosphorylation relative to the 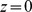 case, but still accounts for a significant increase in phosphorylation relative to the base case 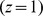. The increase at 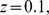 relative to 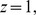 for β, γ, Syk, and LAT phosphorylation are respectively ∼ 2, 3, 6 and 10-fold near the optimal stimulation (Figure 3).

The results show a pattern of phosphorylation for the three proteins, the β ITAM, Syk and LAT as a function lipid raft protection where overall phosphorylation decreases dramatically from 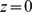 to 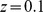 (Figure 3, A, C, and D). Subsequent changes from 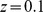 to 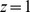 result in more gradual shifts in phosphorylation for these three proteins. This implies that the initial modest access of PTPases into the lipid rafts depletes the pool of phosphorylated proteins substantially, leading to milder effects from the further access of PTPases. In contrast to the three proteins above, the γ ITAM shows a gradual shift in phosphorylation with changing values of the parameter 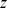 (Figure 3B). This distinction probably arises due to the additional protection of the γ ITAM tyrosines provided by bound Syk SH2 domains as a result of the high concentration of Syk in RBL cells (Table S1). The SH2 domain of Lyn bound to the β ITAM similarly protects the β-ITAM from dephosphorylation but the amount of Lyn in RBL cells available to the receptor is limiting [58]–[60]. In the basal state, approximately 43% of Lyn is phosphorylated on its terminal tyrosine [61] and these Lyns are probably bound intramolecularly to their own SH2 domains and unavailable to interact with the phosphorylated β-ITAM. To account for this, the amount of Lyn available to the receptor is present at much smaller concentration than Syk in the model [41].

### Hapten-induced LAT Dephospohrylation: Resolving Controversies

**Figure 4:**
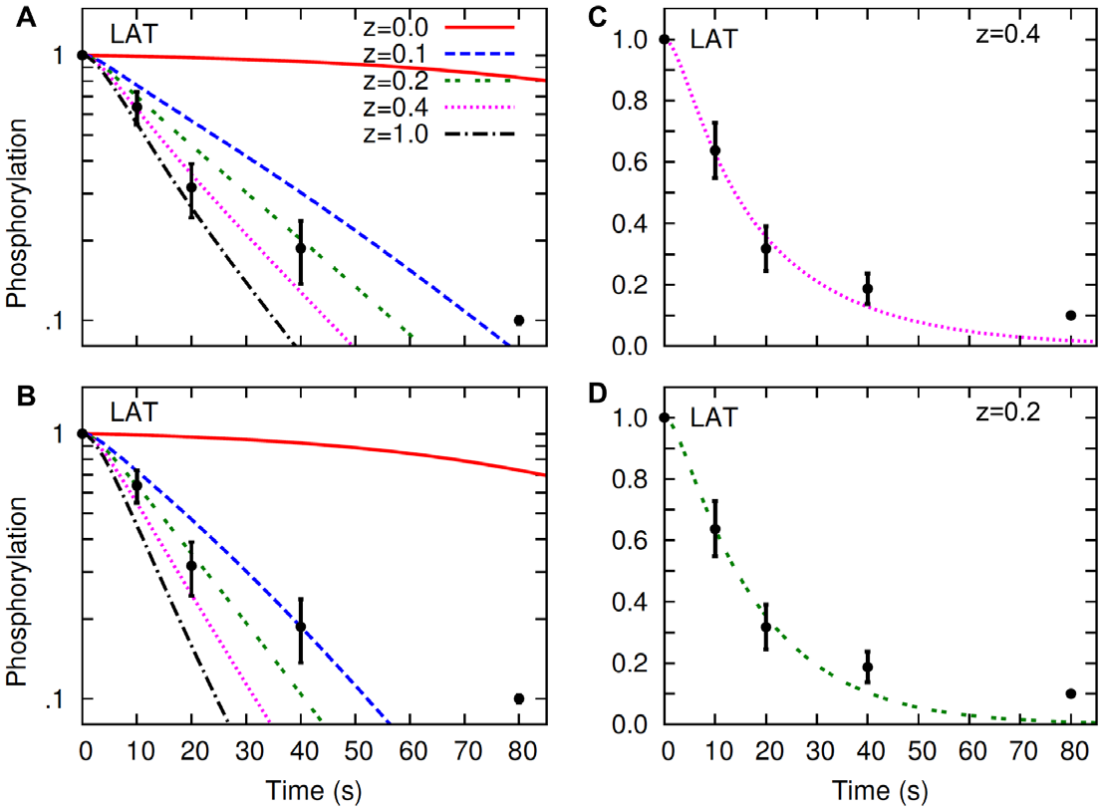
LAT dephosphorylation in the simulated hapten-inhibition experiment. Lines represent the simulation results, and symbols represent the experimental data of Peirce and Metzger [40]. Different lines in panels A and B represent different levels of raft protection as indicated by the parameter 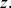 Experimental data and the line at 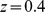 in panel A are re-plotted in linear scale in panel C. Experimental data and the line at 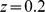 in panel B are re-plotted in linear scale in panel D. The top and the bottom panels represent different values of the receptor-Syk dissociation constant in the model: 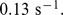 The value taken from Faeder et al. [41], is used in panels A and C, and 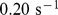 is used in panels B and D. In simulations, steady-state receptor crossliking is induced with a bivalent ligand concentration of 1 nM, and then monovalent hapten is added at 100 µM (Materials and Methods). Time zero indicates the point when hapten is added. In simulations, default values (Table S1) are used for parameters not shown in the figure.

Metzger and colleagues [40], [42] investigated FcεRI and LAT dephosphorylation in RBL cells sensitized with a monoclonal anti-2,4-dinitrophenol (DNP) IgE [62] by inducing receptor aggregation with a multivalent DNP-antigen and then adding an excess of high affinity DNP-hapten in excess to break up the antigen-induced cross-linked receptors. The hapten addition and consequent receptor disaggregation resulted in rapid dephosphorylation of intracellular proteins that were phosphorylated during the receptor aggregation phase of the experiment. They found that FcεRI and LAT dephosphorylated rapidly and with similar kinetics, although the two proteins partition dissimilarly into raft and nonraft parts of the plasma membrane [40]. The experimental data comparing FcεRI and LAT dephosphorylation from Peirce and Metzger [40] is reproduced in Figure 1 (figure 4B in the cited paper). The question we use the model to investigate is, does this dephosphorylation data necessarily require that rafts offer no protection from endogenous PTPases? We used our model to compare the kinetics of dephosphorylation of the FcεRI β and γ ITAMs with their experimental data at different levels of lipid raft protection. We first simulated FcεRI crosslinking in our model with a bivalent ligand (IgE-dimer), and then used a monovalent ligand (hapten) to displace the bivalent ligand from the receptors (Materials and Methods). In the original experiments, a multivalent ligand was used to aggregate IgE, while in the model we used a bivalent ligand, but that difference between experiment and simulation should not matter with respect to the dephospohrylation kinetics once receptors are unaggregated and blocked from reaggregation.

The simulated LAT dephosphorylation kinetics is compared with the experimentally observed LAT dephosphorylation kinetics in Figure 4. The results in the top panels (Figure 4, A and C) and in the bottom panels (Figure 4, B and D) represent different parameter values used in the simulations: Figure 4, A and C were generated by taking parameter values directly from Faeder et al. [41], and Figure 4, B and D were generated with a 35% decreased lifetime of the receptor-Syk complex. The rationale behind the parameter adjustment is discussed in the next section. Briefly, the lifetime was probably overestimated in Faeder et al. [41] because the effect of lipid raft protection was not considered in their model. This lifetime is important because the receptor-Syk complex represents the active pool of Syk that phosphorylates LAT.

When parameters from Faeder et al. [41] were used without the change, the model predicted a moderate level of lipid raft protection, as the simulated kinetics of LAT dephosphorylation was in aggreement with experimentally observed kinetics at 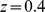 (Figure 4, A and C). When the lifetime of receptor-Syk complex was decreased by 35%, dephosphorylation curves were shifted so that the model predicted a stronger lipid raft protection with 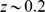 (Figure 4, B and D). The model predicted more pronounced raft protection when the lifetime of receptor-Syk complex was decreased further (result not shown). It is to be noted that the dephosphorylation kinetics of LAT not only depends on the receptor-Syk lifetime, but also on the LAT-Grb2 lifetime, because the LAT phosphotyrosine is protected by the bound Grb2 SH2 domain. Neverthless, by varying the lifetimes of LAT-Grb2 and receptor-Syk binding systematically, similar results can be obtained for reasonable lifetimes for both the complexes.

The above results suggest that the experimentally observed kinetics of LAT dephosphorylation does not contradict raft-mediated protection of LAT from membrane PTPase activities. Depending on the parameter regime in an actual cell, it is possible for LAT to exhibit similar dephosphorylation kinetics as in Peirce and Metzger [40], even in the presence of strong protecton by lipid rafts.

### FcεRI and Syk Dephosphorylation: Effects of SH2 Domain Binding and Lipid Rafts

Faeder et al. [41] used their model to analyze the experimental data in Mao and Metzger [42] that reported the kinetics of FcεRI β and γ dephosphorylation after hapten addition in activated RBL cells. Their analyses revealed that the dephosphorylation kinetics depended on the concentrations of Lyn and Syk, because these proteins bind to phosphotyrosines on β and γ, respectively, via their SH2 domains, and protect these sites from dephosphorylation while bound. They estimated that the concentrations of Lyn and Syk available to interact with the receptor were 28,000 copy/cell and 400,000 copy/cell, respectively. The phosphorylated β-ITAM is protected less efficiently by the smaller available population of Lyn, and dephosphorylated at a faster rate than γ (half-life of phosphorylated β and γ are ∼ 6 s and 11 s, respectively [41]). Faeder et al. [41] obtained the dissociation constants for Lyn and Syk SH2 domain binding to phosphorylated β and γ by fitting their model to the dephosphorylation data in Mao and Metzger [42]. These rate constants were crucial, because their values determined the lifetime of the receptor-Lyn and receptor-Syk complexes, and the lifetimes of these complexes essentially determined the extent of protection the receptor phosphotyrosines received from the protein SH2 domain binding. However, these rate constants may have been underestimated in Faeder et al. [41], because their model did not incorporate the additional effects of lipid raft protection from PTPases. Here, we re-analyze the dephosphorylation kinetics of β and γ by considering lipid raft protection in the membrane.

**Figure 5:**
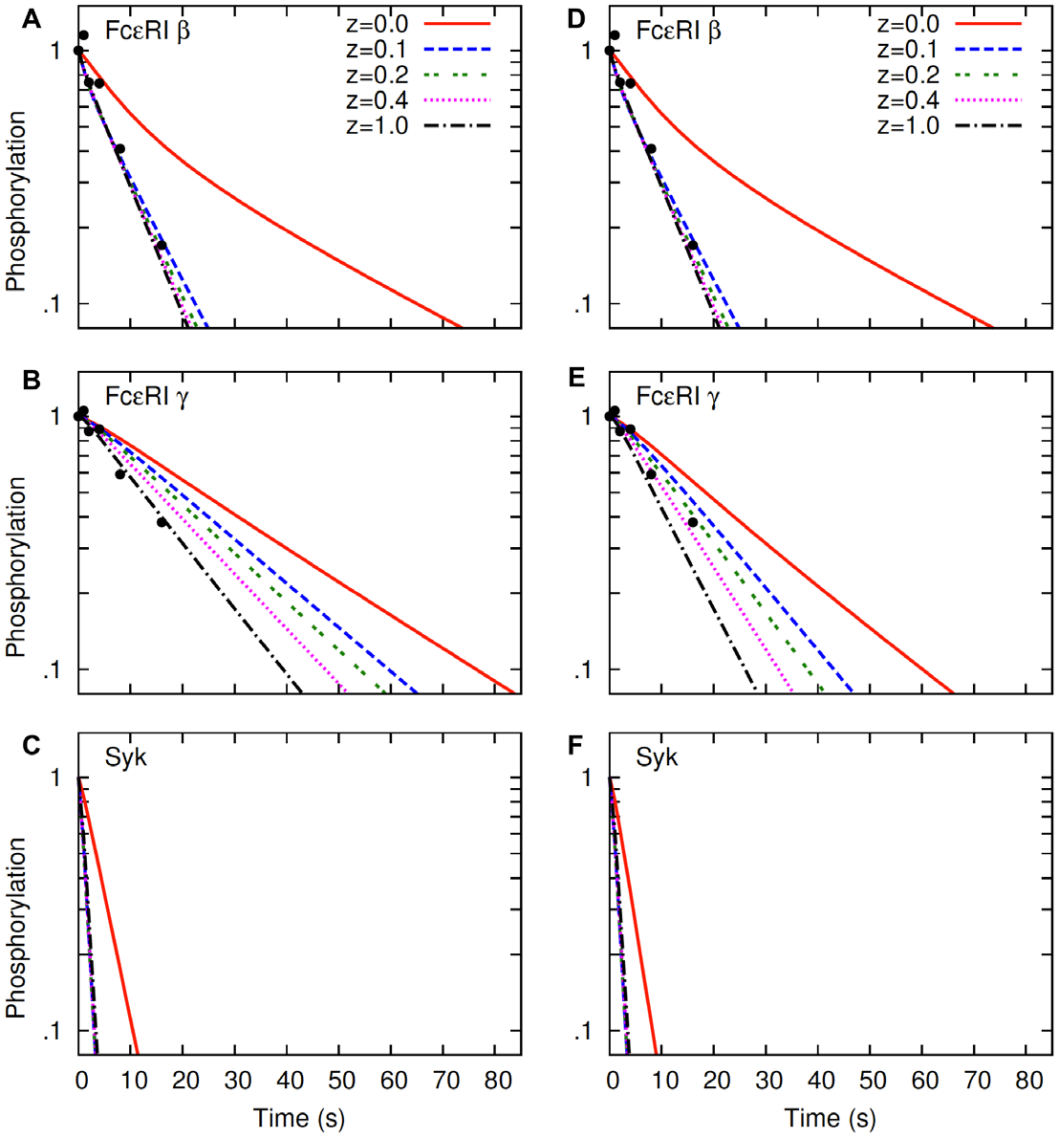
FcεRI β, FcεRI γ and Syk dephosphorylation in the hapten-inhibition experiment. Lines represent the simulation results, and symbols represent the experimental data of Mao and Metzger [42]. In each panel, different lines represent different levels of lipid raft protections as indicated by parameter 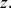 The left panels (A, B, C) represent a receptor-Syk dissociation constant of 0.13 s^−1^ [41], and the right panels (D, E, F) represent a receptor-Syk dissociation constant of 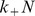 s^−1^. In simulations, default values (Table S1) are used for parameters not shown in the figure.

Figure 5 represents the results from our analyses. The results in the left panels (Figure 5, A*–*C) are produced by using the same Syk dissociation constant as in Faeder et al. [41], 0.13 s^−1^, and the results in the right panels (Figure 5, D–F) are produced by setting this parameter value to 0.2 s^−1^ (50% increase from the original value, which corresponds to a 35% reduction in receptor-Syk lifetime). As expected, the β dephosphorylation kinetics was not changed between the panels as a result of the change in the Syk dissociation constant (Figure 5, A and D). Moreover, lipid rafts essentially had no effect on β dephosphorylation, unless the lipid raft protection was absolute 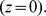 In the range of partial or no lipid raft protection 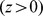, the β dephosphorylation curves all coincided with the experimental data of Mao and Metzger [42] (Figure 5, A and D). In contrast to β, the γ dephosphorylation kinetics was significantly shifted as a result of the relatively minor change in the Syk dissociation constant (Figure 5, B and E). In addition, different levels of lipid raft protection significantly altered the γ dephosphorylation rates (Figure 5, B and E). When the Syk dissociation constant of Faeder et al. [41] was used (Figure 5B), the model predicted essentially no lipid raft protection, because the γ dephosphorylation corresponded to the experimental data at 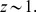 In contrast, when the new Syk dissociation constant was used (Figure 5E), the model predicted significant lipid raft protection, as the simulated kinetics of γ dephosphorylation corresponded to the experimental data at_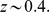._

The differential sensitivity of β and γ to lipid rafts, as observed in Figure 5, reflects the combined effects of lipid raft and SH2 domain-mediated protection. Being available in smaller number, Lyn offered protections to a small fraction of total phosphorylated β inside lipid rafts. Therefore, allowing a small PTPase activity inside lipid rafts could drastically reduce the amount of phosphorylated β, and further access of PTPase activities had little effects (Figure 5, A and D). In contrast, Syk being present in large number, offered protection to most of the phosphorylated γ. Therefore, a more gradual shift was observed in γ dephosphorylation kinetics when different levels of PTPase activities were allowed inside the lipid rafts (Figure 5, B and E).

To explore the effects of lipid rafts on protein phosphorylation in the absence of SH2 domain protection, we analyzed the dephosphorylation kinetics of receptor-bound Syk (Figure 5, C and F). Syk was autophosphorylated in the activation-loop tyrosines upon binding to phosphorylated γ. However, unlike the receptor subunits, in the model Syk phosphorylation sites were not protected by SH2 domains of other proteins. The results show that lipid rafts essentially had no effect on Syk dephosphorylation except in the case of absolute protection (Figure 5, C and F). It is to be noted that Syk binds to phosphorylated γ via tandem SH2 domains with high affinity and therefore the amount of bound Syk should directly correspond to the amount of phosphorylated γ. Despite this dependency of Syk on γ phosphorylation, dephosphorylation kinetics of Syk was more akin to that of β. The similar kinetics of Syk and β dephosphorylation therefore suggests that lipid rafts can barely affect the dephosphorylation kinetics of a protein unless the protein receives an appreciable extent of SH2 domain-mediated protection from other proteins.

### Effects of Lipid Raft Lifetime

**Figure 6:**
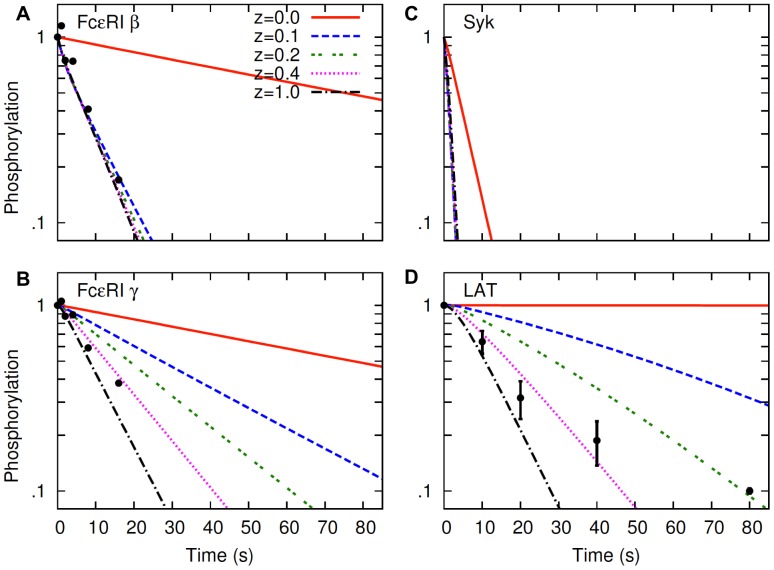
Effects of lipid raft lifetime increase on protein dephosphorylation. The hapten inhibition experiment is simulated with a mean raft lifetime 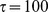 s (instead of 10 s, the default value in the model). With this change in 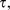 the lumped parameter 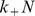 (Eq (1)) is adjusted to keep the partition coefficients of proteins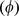 fixed. For other parameters, default values (Table S1) are used. Dephosphorylation kinetics of FcεRI β and γ are shown in panel A and B, respectively. Dephosphorylation kinetics of Syk and LAT are shown in panel C and D, respectively.

**Figure 7:**
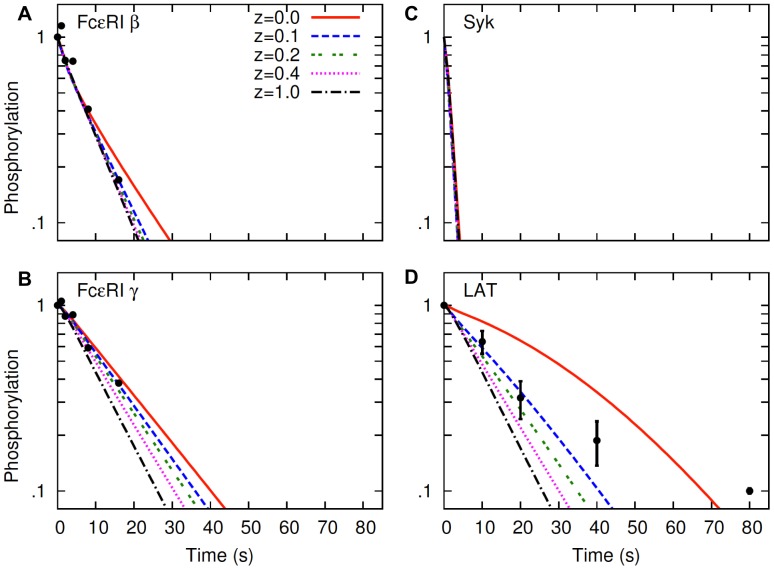
Effects of raft lifetime decrease on dephosphorylation kinetics. The hapten inhibition experiment is simulated with a mean raft lifetime 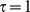 s (instead of 10 s, the default value in the model). With this change in 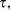 the lumped parameter 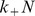 (Eq (1)) is adjusted to keep the partition coefficients of proteins 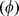 fixed. For other parameters, default values (Table S1) are used. Dephosphorylation kinetics of FcεRI β and γ are shown in panel A and B, respectively. Dephosphorylation kinetics of Syk and LAT are shown in panel C and D, respectively.

In all our simulations we have taken the mean lifetime of lipid rafts 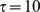 s. Reports of raft lifetimes vary from miliseconds to minutes [3], [63], [64]. To explore how changes in the raft lifetime could affect our analyses, we simulated the dephosphorylation experiments with different raft lifetimes. The results from these simulations are shown in Figure 6 and 7, where the raft lifetime was increaesd and decreased by 10 fold, respecively, relative to the previously used value of 10 s. The Syk dissociation constant used in these (and subsequent) calculations was 0.2 s^−1^, which was also used in Figure 4, B and D, and Figure 5, D–F. Therefore, these new results compare the changes of raft lifetime with respect to the results in Figure 4, B and D, and Figure 5, D–F.

The increase in raft lifetime had small effects on β and Syk dephosphorylation kinetics (Figure 6, A and C, respectively). As before, dephospohrylation rates of these two proteins were insensitive to lipid raft protection, except in the case of absolute protection. In contrast, the increased raft lifetime markedly slowed down the dephosphorylation rates of γ and LAT at all levels of raft protection (Figure 6, B and D, respectively). As is to be expected, as opposed to the increase in raft lifetime, the decrease in lifetime made all proteins dephosphorylate at faster rates (Figure 7). Moreover, the decrease in lifetime led all dephosphorylation curves to approach the base case of no raft protection 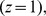 indicating diminished influence of rafts on protein dephoshorylation rates. In particular, for Syk, lipid rafts had no effect at all on its dephosphorylation kinetics at this reduced raft lifetime of 1 s (Figure 7C).

The above effects of raft lifetime reflect the fast intrinsic dephosphorylation rates of proteins in the model, taking one tenth of a second or shorter for the exposed/unprotected phosphotyrosines to dephosphorylate [41]. In the model, the change in raft lifetime does not change the equilibrium partitioning of proteins, meaning the average time a protein spends in raft or nonraft compartment remains the same regardless of raft lifetime. This is because, for every change in raft lifetime 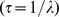, the lumped parameter, 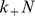 in Eq (1) is adjusted to keep 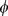 (partition coefficient) constant in the model. However, what is changed due to the change in raft lifetime is the frequency at which proteins alternate between the raft and nonraft compartment. At longer raft lifetimes, proteins alternate between the two compartments less frequently. Under this condition, the depohsphorylation is slowed simply because it takes longer for the first transition (after hapten addition) to occur from raft to nonraft compartment. In contrast, at shorter raft lifetimes, proteins alternate between the two compartments more frequently, and the short exposure to the nonraft compartment in every alternateing cycle contributes to dephosphorylation of the proteins efficiently because dephosphorylation of exposed phosphotyrosines takes place at even shorter time intervals.

The above results in the context of the dephospohrylation experiment represent the effects of 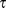 on protein dephosphorylation rates in the absence of re-phosphorylation of proteins by the kinaseses. To see how 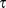 may correlate to protein phosphorylation in the presence of signaling, we looked at the ligand-dose response phosphorylation of FcεRI, Syk, and LAT at 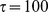 s and 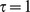 s (Figure S1). As expected, the longer lifetime (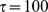 s) enabled stronger lipid raft protection, resulting in more pronounced phosphorylation of proteins at different values of 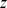 (compare Figure S1, A–D with Figure 3). On the other hand, the shorter lifetime (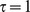 s) also allowed significant raft protection (Figure S1, E–H), contrary to what was expected from the increased dephosphorylation rates in the absence of signaling (Figure 7). With further 10-fold reduction in raft lifetime (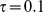 s), the protective effects of lipid rafts on protein phosphorylation were still evident in the dose response plots (result not shown). These results suggest that over a large range of values the lifetime of lipid rafts may not be important for raft-mediated protection of phosphorylated proteins from the PTPases. Rather the main factor in protection is the extent of separation of the proteins and PTPases in the raft and nonraft compartments.

### Lower Limit of Lipid Raft Lifetime: Model Prediction and Theoretical Calculation

The results in the previous section hint at a possible minimum raft lifetime in the model. As seen in Figure 7, with a raft lifetime 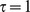 s, the model predicts at all values of 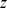 except for 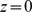 that the dephosphorylation rates are faster than what was observed in the experiment (Figure 7D). With a further 10-fold reduced raft lifetime (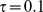 s), the model becomes inconsistent because it predicts faster dephosphorylation rates than the experiment even at 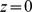 (Figure S2). Raft lifetimes must be 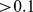 s in the model in order for simulated dephosphorylation curves to be consistent with the experiment.

As the lifetime of the raft is decreased the mean time a protein remains outside the raft must also decrease in order to maintain the correct partitioning, i.e., to keep 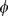 fixed. Shorter raft lifetimes require faster forward rate constants and at some critical raft lifetime, the required forward rate constant will exceed the diffusion limit. The diffusion limited forward rate constant (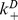) for a protein interacting with a lipid raft depends on the size and the lifetime of the raft, and on the sum of the raft’s and protein’s diffusion coefficients (see Eq. (2)). If rafts diffuse with diffusion coefficients typical of lipids (∼10^−8^ cm^2^/s) [54], a value much higher than diffusion coefficients for surface associated proteins (∼10^−9^–10^−11^ cm^2^/s), then 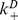 depends only on the raft size and lifetime. Therefore, 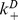 is the same for all cell surface associated proteins outside of rafts. For a raft with a lifetime λ and a protein with a partition coefficient 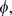 Eq (1) predicts the value of 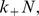 the product of the protein forward rate constant and the surface density of rafts. The larger the rafts, the smaller the value of *N*, as the total amount of membrane in rafts is fixed. Thus, for a given raft lifetime, 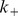 is larger if rafts are larger. This is illustrated in Figure S3 where we show that as the radius of the raft is increased from 100 nm to 1000 nm, which corresponds to *N* going from ∼8000/cell to ∼250/cell, the lifetime of the raft at which the diffusion limit is exceeded goes from ∼0.015 s to 2.0 s. Therefore, for any raft of reasonable size with 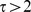 s, 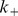 is below the diffusion limit.

### Effects of Mutation in Lyn Palmitoylation on Protein Phosphorylation

An earlier study by Kovarova et al. [29] had demonstrated that mutation of Lyn at the palmitoylation site prevents it from localizing into the lipid rafts, but this does not have significant effects on the phosphorylation of FcεRI, Syk and LAT. To test whether our model is consistent with their observation, we simulated their experiment by allowing differential raft partitioning for the wild type and mutated Lyn, as observed [29]. From their experimental data, we obtained the partition coefficient values, 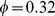 and 0.06 for the wild type and mutated Lyn, respectively (Materials and Methods). It is to be noted that 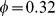 indicates a relatively poor ability of raft association for Lyn, which was expressed in cells exogenously in their experiment. For endogenous Lyn, they reported 55% raft partitioning, and >62% partitioning in RBL cells has been reported by Young et al. [25].

**Figure 8:**
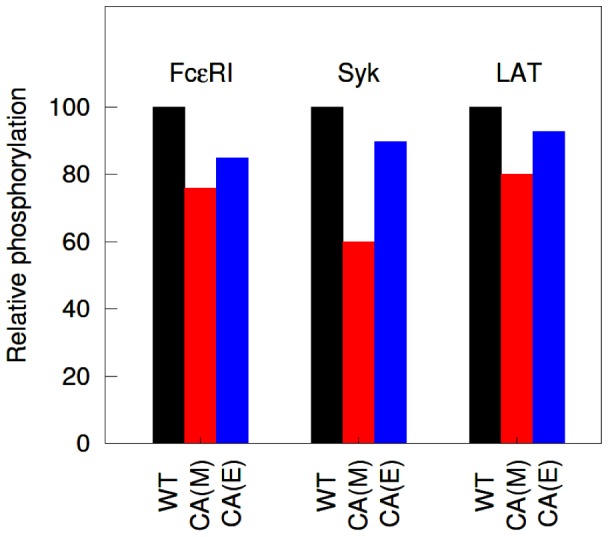
Effects of Lyn palmitoylation mutation on protein phosphorylation. Simulation results are compared with experimental data of Kovarova et al. [29]. In the figure, WT represents wild type Lyn, and CA represents palmitoylation-mutated Lyn. Letter M within parentheses represent model predictions and E represents experimental data. Simulation results presented are for a fixed level of raft protection corresponding to 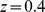. Default values are used for other parameters, as listed in Table S1.

The simulation results and their experimental data are compared in Figure 8. The model predicts approximately 24, 40, and 20% reduction in phosphorylation of FcεRI, Syk, and LAT, respectively, due to the mutation of Lyn (Figure 8). Although these predictions are for a fixed level of raft protection 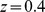 in the model, consistent results can be obtained over a wide range of 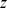 (Figure S4, A–D). Contrary to the model, the experimental data shows a relatively modest ∼15, 10, and 7% reduction in phosphorylation of FcεRI, Syk, and LAT, respectively (Figure 8). Nonetheless, despite this quantitative difference between the model and the experiment, the model consistently suggests that the mutation of Lyn does not entail any remarkable decrease in phosphorylation of the proteins, at least in the context of the experiment.

The results in Figure 8 strictly depend on the partition coefficients being used in the model. With 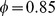 for wild type Lyn (default value used in other calculations, see Table S1), the model predicts a more significant 30–80% decrease in phosphorylation of FcεRI, Syk, and LAT as a result of the mutation of Lyn (Figure S4, E–H).

## Discussion

In this study we have extended our previous model of mast cell signaling [41] to include LAT, Grb2 and lipid rafts and used the model to answer the following question: in RBL cells can lipid rafts offer protection from PTPases that results in enhanced signaling [19], [24], [25], and still exhibit the receptor and LAT dephosphorylation kinetics observed by Peirce and Metzger [40]? To answer this question we have modeled the plasma membrane as consisting of a single population of rafts embedded in a homogeneous membrane of disorder lipid (the nonraft compartment). Within a raft PTPase activity is reduced, presumably through the partial exclusion of PTPases. Aggregated receptors, LAT and Lyn partition to rafts while unaggregated receptors are indifferent to rafts. Two cytosolic proteins, the protein tyrosine kinase Syk and the adapter Grb2 complete the list of proteins in the model (Figure 2). When an SH2 domain is bound to a phosphotyrosine, the phosphotyrosine is protected from dephosphorylation [41]. In the model, such protection occurs when Syk binds through its SH2 domains to phosphorylated ITAMs on receptor γ-chains, when Lyn binds through its SH2 domain to phosphorylated ITAMs on receptor β-chains, and when Grb2 binds to phosphorylated Grb2 binding sites on LAT. In the dephosphorylation experiments we analyze, these reversible binding reactions and the rates of dephosphorylation of naked phosphotyrosines in and out of rafts determine the kinetics of dephosphorylaton. We assume that the rafts are transient, forming and breaking up with some average lifetime, but with the total number of rafts remaining constant.

Peirce and Metzger [40] monitored the dephosphorylation kinetics of unaggregated receptors (nonraft associated) and LAT (raft-associated) and found them to decay at almost identical rates (Figure 1). From this they concluded that since a raft and nonraft protein dephosphorylated with similar kinetics, rafts offered no protection from PTPases. Our model predictions show that if rafts offer absolute protection from dephosphorylation, LAT dephosphorylation would indeed be much slower than receptor dephosphorylation (Figure 4). LAT would still dephosphorylate since rafts have finite lifetimes and therefore no raft-associated protein would spend all its time in a raft. However, the model also predicts that rafts can offer substantial protection from dephosphorylation and yet receptors and LAT can dephosphorylate with similar kinetics. Figure 4B shows that with a rate of dephosphorylation that is five times slower in the raft than out 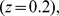 the predicted kinetics of LAT dephosphorylation is in agreement with the experiments of Peirce and Metzger [40]. Comparing model predictions to experiment, we conclude that these dephosphorylation data rule out rafts on RBL cells that offer absolute protection from dephosphorylation to LAT and presumably, other raft associated proteins, but that these experiments rule out little else. Rafts can offer substantial protection from dephosphorylation to LAT and still be consistent with the Peirce and Metzger experiments [40].

In the model we assume a distribution of identical rafts where each raft takes up an area of 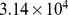 nm^2^. If the raft were circular this would correspond to a raft of 100 nm radius. In the literature a broad size distribution of rafts has been reported, ranging from a few nanometers to one micrometer [1]. For model predictions the geometry and size of the raft are unimportant as long as the forward rate constant for a surface protein interacting with a raft is reaction limited. We show for any reasonable size raft whose lifetime is greater than a few seconds, 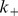 is reaction limited. Rather, it is the total area that the lipid rafts occupy in the plasma membrane that is a key parameter in the model. We have taken this aggregated area (raft compartment) to be 30% of the plasma membrane for an RBL cell [46].

Smaller lipid rafts may be present in the plasma membrane, but we are interested only in those lipid rafts that might be actively involved in cell signaling. The ability to mediate cell signaling imposes a restriction on the minimum size of the rafts. A lipid raft smaller than a certain size would be unable to accommodate a reasonable number of signaling proteins within it to induce signaling. A raft of 100 nm radius corresponds to a total of about 8,000 lipid rafts for an RBL cell of surface area 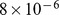 cm^2^ [65] and a raft compartment covering 30% of the surface area [46]. Wofsy et al. [58] estimated that there are about 28,000 Lyn available to interact with the receptor. This gives an average number of active Lyn per raft of ∼ 3.5 molecules. A lipid raft much smaller than the assumed size in our model would be compromised in its ability to induce signaling due to the scarcity of the signaling proteins within it.

In the model, we do not explicitly incorporate the phosphorylation-induced regulation of Lyn kinase activity. Full catalytic activation of Lyn requires Lyn, in its fully open configuration, to be phosphorylated on a tyrosine residue in its activation loop [66]. This active state typically represents a small fraction of the total Lyn present in a cell [58]. Following the original FcεRI signaling model of Faeder et al. [41], we only include this active fraction as the effective Lyn in the system, and do not consider the explicit autophosphorylation/dephosphorylation of the activation loop tyrosine or the terminal tyrosine of Lyn. Because of the higher PTPase activity outside the rafts we expect the lifetime of fully active Lyn to be reduced in the nonraft region, but this dephosphorylation of Lyn is not in the model. To compensate we take the activity of Lyn outside the rafts to be 5-fold lower than inside the rafts, as reported in (24). Since in our model ∼85% of the total Lyn, FcεRI-dimer and LAT remain dynamically partitioned into the raft compartment, most of the activities of the Lyn kinase occur inside the raft compartment.

The lifetime of lipid rafts in the plasma membrane is of particular interest since it is thought to be an important criterion for lipid raft function in signal transduction and other cellular processes [63]. Experiments have yielded a broad range of raft lifetime, from milliseconds to minutes [3], [63], [64]. Perhaps surprisingly, our model predicts that the lifetime of lipid rafts is not important for raft-mediated protection against the PTPases. The model predicts that as long as the total amount of rafts and the partitioning of proteins in the membrane are fixed, significant raft protection can be achieved over a wide range of raft lifetimes.

In the model, we assume LAT, Lyn and aggregated receptors localize in the same raft compartment. Detergent extraction experiments have identified LAT predominantly localized in lipid rafts [9], [30], [47], whereas electron microscoy has revealed that LAT and aggregated FcεRI are spatially resolved in the cell membrane, indicating LAT may partition outside the raft domains [33]. We have focused here on a specific set of experimental data that revealed rapid dephosphorylation of LAT upon hapten-disruption of the FcεRI aggregates [40], indicating intimate communication between the proteins. We presume that such intimate communication required at least brief colocalization of LAT and the antigen-crosslinked FcεRI complexed with Syk. Recent experiments using cross-correlation image analysis support the picture that LAT and Lyn co-localize in RBL cells [49].

We investigated the effects of Lyn mutation at its palmitoylation site, which is required for its raft localization. An earlier study [29] suggested that raft localization of Lyn is not important for the initiation of FcεRI signaling, as mutation of its palmitoylation site did not significantly decrease phosphorylation of FcεRI, Syk, and LAT in their experiment. By simulating their experiment, we show that the modest effect could arise as a result of the low raft partitioning (32%) of the exogenously expressed Lyn in their experiment. The model predicts that, in the case of higher raft partitioning of Lyn, as typically observed in the RBL cells, the mutation of Lyn could significantly decrease phosphorylation of these proteins.

In the model, lipid rafts are dynamic entities with finite lifetimes that appear and disappear randomly on the cell surface, but whose size remains fixed during their lifetime. However, several studies have indicated that the formation of lipid rafts is a dynamic process induced by ligand-crosslinking of some lipids and proteins in the cell membrane [5], [6], [67]. These studies have suggested that proteins, such as FcεRI and other immune receptors, have small amounts of raft components surrounding them. Upon ligand crosslinking, these proteins form aggregates, which bring together the protein-associated raft components that coalesce to form the larger patches of the raft domains. Extending the model to include the kinetics of raft formation is an alternative problem, but it is not clear that there is sufficient quantitative data to test such a model at present.

As with almost all models, there is much that has been omitted from this model. The inhomogeneities in the plasma membrane are much more complex than the model we have used to describe the plasma membrane [68]. As a result, in its present form, the model is limited in the questions about lipid rafts that it can be used to investigate. We have designed the model to answer a specific question concerning a set of dephosphorylation experiments: Do theses experiments [40] rule out the possibility that rafts offer protection from PTPases? Although the model is quite simple in its structure, it is sufficiently detailed to answer this question. Comparison of the model with experiment leads us to conclude that these dephosphorylation experiments do not rule out the possibility that rafts offer protection from PTPases.

## Supporting Information

Figure S1
**Effects of raft lifetime increase or decrease on protein phosphorylation.** The figure shows similar ligand dose response plots as in Fig. 3 (main text), but at raft lifetime 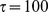 s (left panels A–D) and 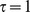 s (right panels E–H) (instead of the default lifetime 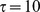 s in Fig. 3). Except for 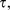 other parameter are the same as in Fig. 3 (Table S1).(TIFF)

Figure S2
**Protein dephosphorylation in the simulated hapten-inhibition experiment.** A 100-fold shorter raft lifetime 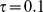 s (default lifetime 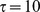 s) is used. The figure should be compared with Figs. 6 and 7 (main text), where relative longer raft lifetimes (100 s and 1 s, respectively) respectively are used. Except for 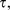 other parameter values used are the same as in Figs. 6 and 7 (Table S5).(TIFF)

Figure S3
**Theoretical bound for the shortest raft lifetime 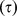.** The two dimensional forward rate constants of individual proteins 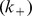
from Eq. (1) (Materials and Methods) and the diffusion-limited forward rate constant 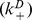 from Eq. (2) (Materials and Methods) are plotted as functions of 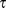 for the two cases of distinct raft size. (A) The plot shows the case where rafts are of 100 nm radius (default raft size in the model). The intersection where 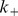 (for Lyn, LAT or receptor dimer) first crosses 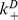 represents the shortest permissible raft lifetime in the model, which is ∼ 0.015 s. (B) the plot shows the case where rafts are of 1,000 nm radius. The intersection of curves suggests the shortest permissible raft lifetime for this raft size to be ∼1.5 s.(TIFF)

Figure S4
**Effects of Lyn palmitoylation mutation on protein phosphorylation.** Relative phosphorylation of FcεRI β, FcεRI γ, Syk, and LAT by wild type Lyn (WT) and mutated Lyn (Lyn-CA) are shown as function of lipid raft protection 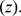 In the left panels (A–D) simulations are carried out with partition coefficients 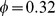 for the wild type Lyn, and 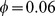 for the mutated Lyn, and in the right panels (E–H) simulations are carried out with 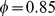 for the wild type Lyn, and 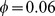 for the mutated Lyn. Other parameter values used in the simulations are listed in Table S1.(TIFF)

Table S1 **Model parameter values.**
(DOCX)

Text S1 **BioNetGen model input file (LipidRaft.bngl).**
(DOCX)
