# Correction: A Mechanistic Model of Early FcεRI Signaling: Lipid Rafts and the Question of Protection from Dephosphorylation

**DOI:** 10.1371/annotation/4aae3d8f-f237-41ed-ad89-25d91160163b

**Published:** 2013-08-23

**Authors:** Dipak Barua, Byron Goldstein

A funding organization and grant were incorrectly omitted from the Funding Statement.

The Funding Statement should read: "Research reported in this publication was supported by the National Institute of General Medical Sciences of the National Institutes of Health under award number R37-GM035556, The SpatioTemporal Modeling Center at the University of New Mexico under award number P50:GM065794 and the Department of Energy through contract DE-AC52-06NA25396. The funders had no role in study design, data collection and analysis, decision to publish, or preparation of the manuscript." 

