# Supplementary figures and images for: A Mechanistic Model of Early FcεRI Signaling: Lipid Rafts and the Question of Protection from Dephosphorylation
Source: PLoS One. 2012 Dec 17;7(12):e51669. doi: 10.1371/journal.pone.0051669 (PMC3524258; doi:10.1371/journal.pone.0051669)

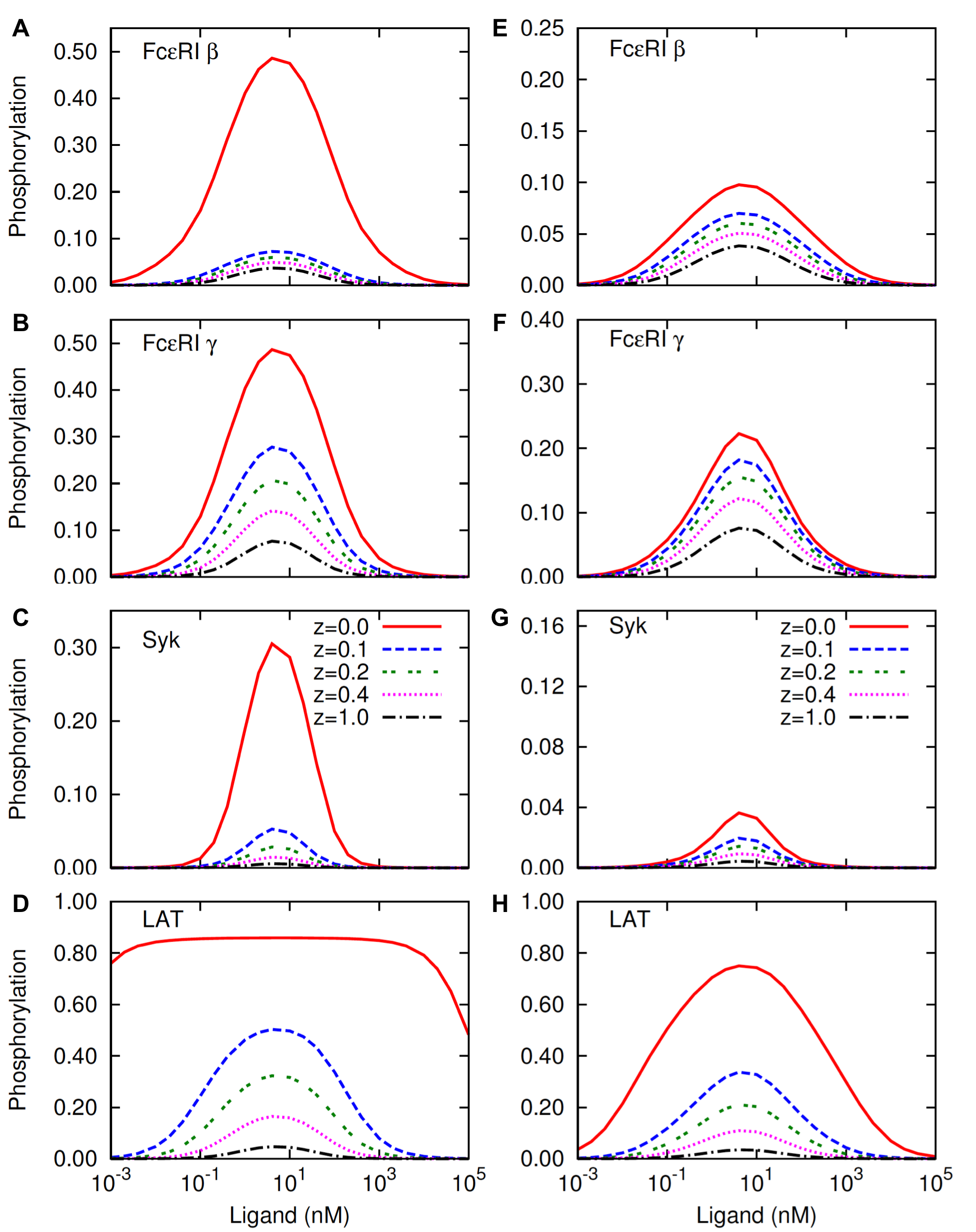

Supplement: Figure S1 — Effects of raft lifetime increase or decrease on protein phosphorylation. The figure shows similar ligand dose response plots as in Fig. 3 (main text), but at raft lifetime s (left panels A–D) and s (right panels E–H) (instead of the default lifetime s in Fig. 3). Except for other parameter are the same as in Fig. 3 (Table S1). (TIFF) [file pone.0051669.s001.tiff]

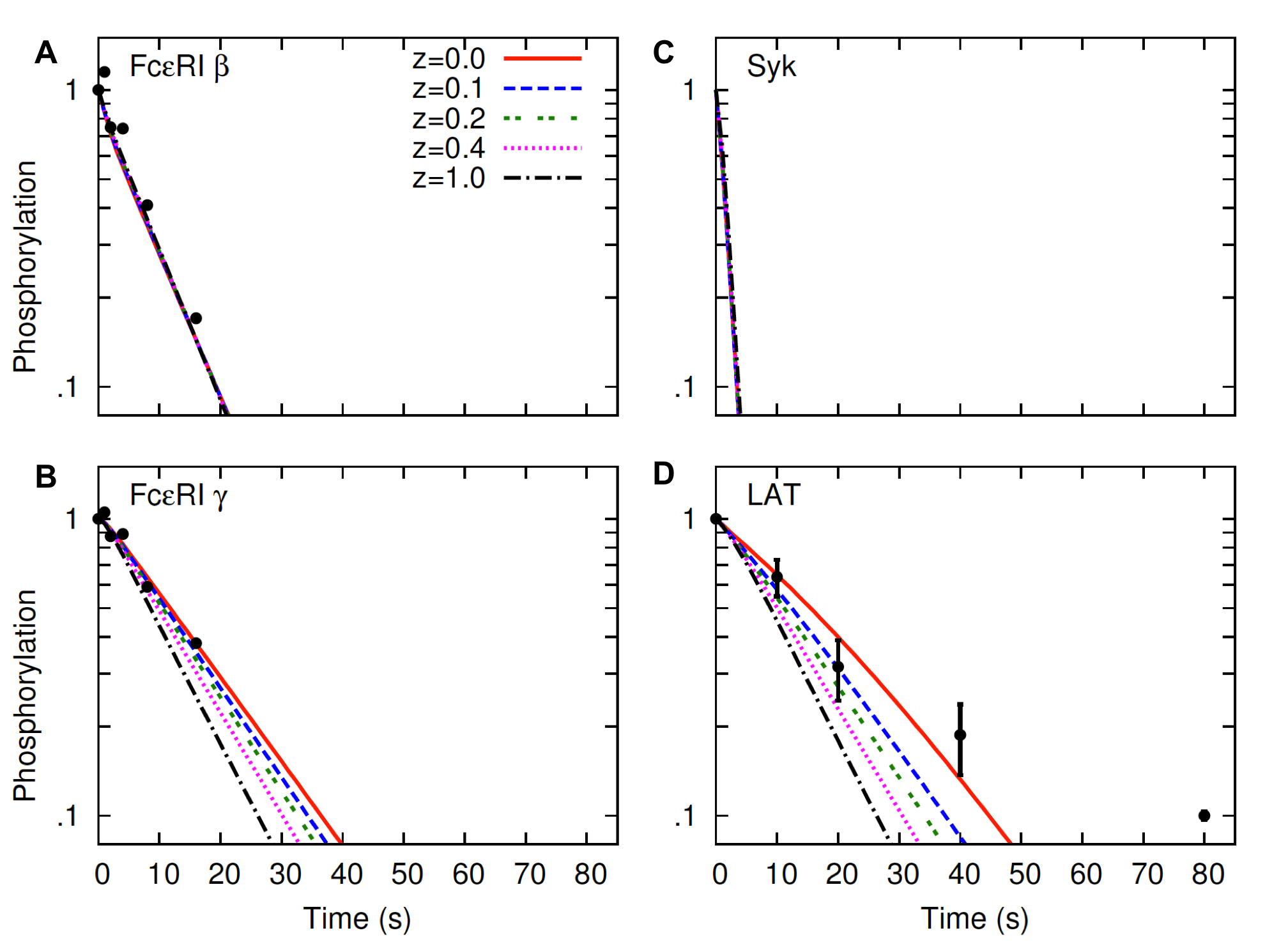

Supplement: Figure S2 — Protein dephosphorylation in the simulated hapten-inhibition experiment. A 100-fold shorter raft lifetime s (default lifetime s) is used. The figure should be compared with Figs. 6 and 7 (main text), where relative longer raft lifetimes (100 s and 1 s, respectively) respectively are used. Except for other parameter values used are the same as in Figs. 6 and 7 (Table S5). (TIFF) [file pone.0051669.s002.tiff]

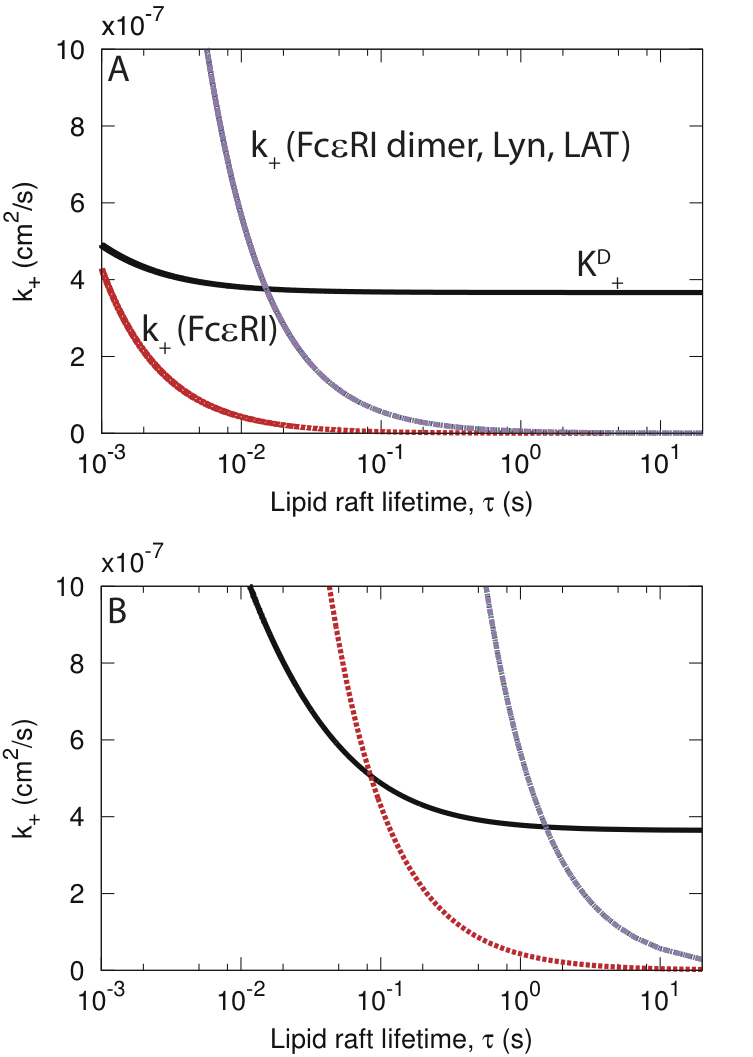

Supplement: Figure S3 — Theoretical bound for the shortest raft lifetime . The two dimensional forward rate constants of individual proteins from Eq. (1) (Materials and Methods) and the diffusion-limited forward rate constant from Eq. (2) (Materials and Methods) are plotted as functions of for the two cases of distinct raft size. (A) The plot shows the case where rafts are of 100 nm radius (default raft size in the model). The intersection where (for Lyn, LAT or receptor dimer) first crosses represents the shortest permissible raft lifetime in the model, which is ∼ 0.015 s. (B) the plot shows the case where rafts are of 1,000 nm radius. The intersection of curves suggests the shortest permissible raft lifetime for this raft size to be ∼1.5 s. (TIFF) [file pone.0051669.s003.tiff]

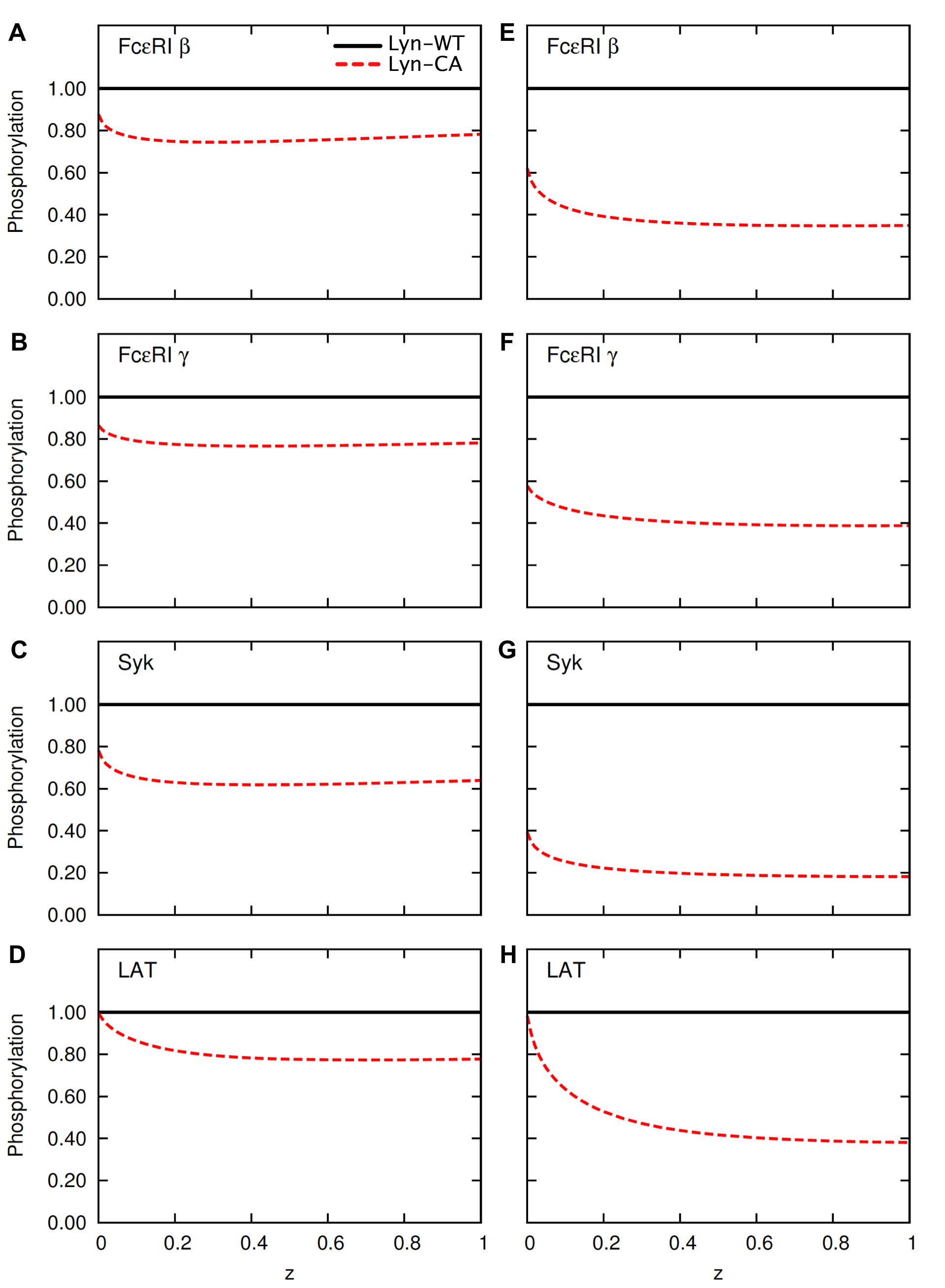

Supplement: Figure S4 — Effects of Lyn palmitoylation mutation on protein phosphorylation. Relative phosphorylation of FcεRI β, FcεRI γ, Syk, and LAT by wild type Lyn (WT) and mutated Lyn (Lyn-CA) are shown as function of lipid raft protection In the left panels (A–D) simulations are carried out with partition coefficients for the wild type Lyn, and for the mutated Lyn, and in the right panels (E–H) simulations are carried out with for the wild type Lyn, and for the mutated Lyn. Other parameter values used in the simulations are listed in Table S1. (TIFF) [file pone.0051669.s004.tiff]
