# Supplementary material for: A Mechanistic Model of Early FcεRI Signaling: Lipid Rafts and the Question of Protection from Dephosphorylation
Source: PLoS One. 2012 Dec 17;7(12):e51669. doi: 10.1371/journal.pone.0051669 (PMC3524258; doi:10.1371/journal.pone.0051669)
Supplement: Table S1 — Model parameter values. (DOCX) [file pone.0051669.s005.docx]

**Table S1. Model parameter values**

| **Parameter** | **Value** | **Comment** |
| --- | --- | --- |
| **Cell membrane** |  |  |
| Total membrane area, *S_m_* | 8 × 10^-6^ cm^2^ | Ref. ([1](#_ENREF_1)) |
| Aggregated raft area, *S_r_* | 0.3S_m_ cm^2^ | Lipid rafts occupy 30-40% of the cell membrane area in RBL cells ([2](#_ENREF_2)). |
| **Lipid rafts** |  |  |
| Radius, *a* | 100 nm | This assumption for individual raft dimension is not important in the model; a different raft size does not alter the results presented. |
| Density, *N* | 10^9^ /cm^2^ | Based on raft size (*a* =100 nm) and aggregated raft area  (0.3S_m_ cm^2^) |
| Mean lifetime, *τ* | 1-100 s | Default value is 10 s |
| **Protein partitioning** |  |  |
| Receptor monomer, **** | 0.30 | Since receptor monomers distribute randomly in the plasma membrane ([3](#_ENREF_3), [4](#_ENREF_4)){Field, 1997 #93}, ****implies 30% of them partitions into the raft compartment, which represents 30% of the cell membrane. |
| Receptor dimer, **** | 0.85 | Estimated from Ref. ([5](#_ENREF_5)) (Materials and Methods). |
| Lyn, **** | 0.85 | Assumed to be the same as receptor dimer. In Ref. ([6](#_ENREF_6)), > 62% raft partitioning of Lyn has been reported. |
| LAT, **** | 0.85 | Assumed the same as receptor dimer. LAT taken to be as a localized protein based on ([7-9](#_ENREF_7)) (Materials and Methods). |
| **$k_{+,R}$** | **** | Two-dimensional forward rate constant for lipid raft – FcεRI monomer association (Eq (1)). |
| **$k_{+,D}$** | **** | Two-dimensional forward rate constant for lipid raft - FcεRI dimer association (Eq (1)). |
| **$k_{+,L}$** | **** | Two-dimensional forward rate constant for lipid raft - Lyn association (Eq (1)). |
| **$k_{+,T}$** | **** | Two-dimensional forward rate constant for lipid raft - LAT association (Eq (1)). |
| $k_{-R}, k_{-D}, k_{-L}, k_{-T}$ | **$1/\tau$** | Reverse constants (proteins dissociation from lipid rafts, equal to lipid raft turn over rate, **$\lambda=1/\tau$**) |
| **Concentrations** |  |  |
| FcεRI, *Rec_T_* | 4 × 10^5^/cell | Estimated number of FcεRI in RBL cells, Ref. ([10](#_ENREF_10)). |
| Lyn, *Lyn_T_* | 2.8 × 10^4^/cell | Available Lyn is ~ 0.07 × *R*_T_ ([10](#_ENREF_10), [11](#_ENREF_11)). |
| Syk, *Syk_T_* | 4 × 10^5^/cell | Ref. ([10](#_ENREF_10)). |
| LAT, *LAT_T_* | 1 × 10^6^/cell | Ref. ([12](#_ENREF_12)). |
| Grb2, *Grb_T_* | 4 × 10^5^/cell | Ref. ([12](#_ENREF_12)) |
| **Receptor-ligand binding** |  |  |
| *k_on_* | 5 × 10^7^ M^-1^s^-1^ *^a^*  (8.125 × 10^-8^ molecules^-1^s^-1^) | Ligand binding to FcεRI from solution. |
| *k_x_* | 4 × 10^-11^ cm^2^ s^-1^  (5 × 10^-6^ molecules^-1^s^-1^) | Ligand binding to FcεRI from membrane. |
| *k_off_* | 0.5 s^-1^ | Ligand dissociation |
| **Reeptor-hapten binding** |  |  |
| *k_on,h_* | 2.6 × 10^7^ M^-1^s^-1^ *^a^*  (4.3 × 10^-8^ molecules^-1^s^-1^) | Hapten binding to FcεRI (from solution) (Ref. ([13](#_ENREF_13))). |
| *k_off,h_* | 0.019 s^-1^ | Hapten dissociation (Ref. ([13](#_ENREF_13))). |
| **Receptor-Lyn binding** |  |  |
| *l_f_* | 4 × 10^-10^ cm^2^s^-1^  (5 × 10^-5^ molecules^-1^s^-1^) | Lyn association with unphosphorylated/phosphorylated β ITAM via unique/SH2 domain. Parameter value is estimated in Ref. ([10](#_ENREF_10)). |
| *l_r,1_* | 20 s^-1^ | Lyn unique domain dissociation. Parameter value is estimated in Ref. ([10](#_ENREF_10)). |
| *l_r,2_* | 0.12 s^-1^ | Lyn SH2 domain dissociation. Parameter value is estimated in Ref. ([10](#_ENREF_10)). |
| **Receptor-Syk binding** |  |  |
| *s_f_* | 5 × 10^7^ M^-1^s^-1^ *^b^*  (6 × 10^-5^ molecules^-1^s^-1^) | Syk association with phosphorylated γ ITAM. Parameter value is estimated in Ref. ([10](#_ENREF_10)). |
| *s_r_* | 0.20 s^-1^ | Syk dissociation. Parameter value estimated in Ref. ([10](#_ENREF_10)) is 0.13 s^-1^ *^c^*. |
| **LAT-Grb2 association** |  |  |
| *g_f_* | 10^6^ M^-1^s^-1^ *^b^*  (1.25 × 10^-6^ molecules^-1^s^-1^) | Grb2 association with phosphorylated LAT (Ref. ([12](#_ENREF_12))). |
| *g_r_* | 0.30 s^-1^ | Grb2 dissociation (Ref. ([12](#_ENREF_12))). |
| **Phosphorylation** |  |  |
| *p_Lβ1,o_* | 30 s^-1^ | FcεRI β phosphorylation mediated by unique domain-bound Lyn in raft regions. Parameter value based on Ref. ([10](#_ENREF_10)). |
| *p_Lβ1,d_* | 6 s^-1^ | Same interaction as above in nonraft regions. Lyn kinase activity reduced by 5-fold ([14](#_ENREF_14)). |
| *p_Lβ2,o_* | 100 s^-1^ | FcεRI β phosphorylation mediated by SH2 domain-bound Lyn in raft regions. Parameter value based on Ref. ([10](#_ENREF_10)). |
| *p_Lβ2,d_* | 20 s^-1^ | Same interaction as above in nonraft regions. Lyn kinase activity reduced by 5-fold ([14](#_ENREF_14)). |
| *p_Lγ1,o_* | 1 s^-1^ | FcεRI γ phosphorylation mediated by unique domain-bound Lyn in raft regions. Parameter value based on Ref. ([10](#_ENREF_10)). |
| *p_Lγ1,d_* | 0.2 s^-1^ | Same interaction as above in nonraft regions. Lyn kinase activity reduced by 5-fold ([14](#_ENREF_14)). |
| *p_Lγ2,o_* | 3 s^-1^ | FcεRI γ phosphorylatoin mediated by SH2 domain-bound Lyn. Parameter value based on Ref. ([10](#_ENREF_10)). |
| *p_Lγ2,d_* | 0.6 s^-1^ | Same interaction as above in nonraft regions. Lyn kinase activity reduced by 5-fold ([14](#_ENREF_14)). |
| *p_ss,1_* | 100 s^-1^ | Syk autophosphorylation mediated by activation loop unphosphorylated Syk. Parameter value based on Ref. ([10](#_ENREF_10)). |
| *p_ss,2_* | 200 s^-1^ | Syk autophosphorylation mediated by activation loop phosphorylated Syk. Parameter value based on Ref. ([10](#_ENREF_10)). |
| *p_sl_* | 5 × 10^7^ M^-1^s^-1^  (6 × 10^-5^ molecules^-1^s^-1^) | LAT phosphorylation by receptor-bound Syk  Equivalent maximum first order rate constant,  *p_sl_ S_T_*  = 24 s^-1^. |
| **Dephosphorylation** |  |  |
| *d_β_* , *d_γ_* , *d_s_* | ******s^-1^ | First order dephosphorylation rate constants for β, γ, and Syk. Estimated 20 s^-1^ in Ref. ([10](#_ENREF_10))*^d^*. |
| *d_l_* | ******s^-1^ | First order dephosphorylation rate constant for LAT used in this study. |

*^a^*Cell density assumed to be 10^6^ cells/ml ([10](#_ENREF_10)).

*^b^*Cell volume assumed to be 1.4 × 10^-9^ ml ([10](#_ENREF_10)).

*^c^*Faeder et al. ([10](#_ENREF_10)) estimated **$k_{off}$**= 0.13 s^-1^ for receptor-Syk binding by fitting their model to γ ITAM dephosphorylation kinetics in Mao and Metzger ([15](#_ENREF_15)). Here, this value is adjusted to 0.2 s^-1^ (1.5-fold increase) to take into account the additional protective effects of lipid rafts on γ phosphorylation.

*^d^*Values from Faeder et al. ([10](#_ENREF_10)) are multiplied by**** **$z=1$** in the nonraft compartment, and ****in the raft compartment.

**References**

1. Rosenbluth, M. J., W. A. Lam, and D. A. Fletcher. 2006. Force microscopy of nonadherent cells: a comparison of leukemia cell deformability *Biophys J* 90:2994-3003.

2. Holowka, D., and B. Baird. 2001. FcεRI as a paradigm for a lipid raft-dependent receptor in hematopoietic cells *Semin Immunol* 13:99-105.

3. Field, K. A., D. Holowka, and B. Baird. 1995. FcεRI-mediated recruitment of p53/56lyn to detergent-resistant membrane domains accompanies cellular signaling *Proc Natl Acad Sci U S A* 92:9201-9205.

4. Field, K. A., D. Holowka, and B. Baird. 1997. Compartmentalized activation of the high affinity immunoglobulin E receptor within membrane domains *J Biol Chem* 272:4276-4280.

5. Kovarova, M., P. Tolar, R. Arudchandran, L. Draberova, J. Rivera, and P. Draber. 2001. Structure-function analysis of Lyn kinase association with lipid rafts and initiation of early signaling events after Fcε receptor I aggregation *Mol Cell Biol* 21:8318-8328.

6. Young, R. M., X. Zheng, D. Holowka, and B. Baird. 2005. Reconstitution of regulated phosphorylation of FcεRI by a lipid raft-excluded protein-tyrosine phosphatase *J Biol Chem* 280:1230-1235.

7. Arudchandran, R., M. J. Brown, M. J. Peirce, J. S. Song, J. Zhang, R. P. Siraganian, U. Blank, and J. Rivera. 2000. The Src homology 2 domain of Vav is required for its compartmentation to the plasma membrane and activation of c-Jun NH(2)-terminal kinase 1 *J Exp Med* 191:47-60.

8. Zhang, W., R. P. Trible, and L. E. Samelson. 1998. LAT palmitoylation: its essential role in membrane microdomain targeting and tyrosine phosphorylation during T cell activation *Immunity* 9:239-246.

9. Tanimura, N., M. Nagafuku, Y. Minaki, Y. Umeda, F. Hayashi, J. Sakakura, A. Kato, D. R. Liddicoat, M. Ogata, T. Hamaoka, and A. Kosugi. 2003. Dynamic changes in the mobility of LAT in aggregated lipid rafts upon T cell activation *J Cell Biol* 160:125-135.

10. Faeder, J. R., W. S. Hlavacek, I. Reischl, M. L. Blinov, H. Metzger, A. Redondo, C. Wofsy, and B. Goldstein. 2003. Investigation of early events in FcεRI-mediated signaling using a detailed mathematical model *J Immunol* 170:3769-3781.

11. Wofsy, C., C. Torigoe, U. M. Kent, H. Metzger, and B. Goldstein. 1997. Exploiting the difference between intrinsic and extrinsic kinases: implications for regulation of signaling by immunoreceptors *J Immunol* 159:5984-5992.

12. Nag, A., M. I. Monine, J. R. Faeder, and B. Goldstein. 2009. Aggregation of membrane proteins by cytosolic cross-linkers: theory and simulation of the LAT-Grb2-SOS1 system *Biophys J* 96:2604-2623.

13. Erickson, J., B. Goldstein, D. Holowka, and B. Baird. 1987. The effect of receptor density on the forward rate constant for binding of ligands to cell surface receptors *Biophys J* 52:657-662.

14. Young, R. M., D. Holowka, and B. Baird. 2003. A lipid raft environment enhances Lyn kinase activity by protecting the active site tyrosine from dephosphorylation *J Biol Chem* 278:20746-20752.

15. Mao, S. Y., and H. Metzger. 1997. Characterization of protein-tyrosine phosphatases that dephosphorylate the high affinity IgE receptor *J Biol Chem* 272:14067-14073.
