# Supplementary material for: A Mechanistic Model of Early FcεRI Signaling: Lipid Rafts and the Question of Protection from Dephosphorylation
Source: PLoS One. 2012 Dec 17;7(12):e51669. doi: 10.1371/journal.pone.0051669 (PMC3524258; doi:10.1371/journal.pone.0051669)
Supplement: Text S1 — BioNetGen model input file (LipidRaft.bngl). (DOCX) [file pone.0051669.s006.docx]

**Text S1: BioNetGen Model Input File (LipidRaft.bngl)**

**# Notes:**

#The model represents (1/N) of a cell, where N=8,000, number of rafts per cell.

# To make it represent the entire cell, replace N with 1, wherever it is used to scale the parameter values.

# State (s~o) represents raft-localized state of a protein, and state (s~d) represents nonraft state

# of a protein.

**begin parameters**

# Concentrations

LigT 6.0e5 # Ligand; Equivalent to 1 nM

RecT 400000/N # Receptor (FceRI)

LynT 28000/N # Lyn

SykT 400000/N # Syk

LATT 1000000/N # LAT

GrbT 400000/N # Grb2

#HapT 60000000000 # Monovalent hapten;

kon 8.125e-8 # Ligand binding to receptor from solution

kx N*5e-6 # Ligand binding to receptor from membrane (receptor crosslinking)

koff 0.5 # Ligand dissociation

kon_h 4.3e-8 # Hapten binding to receptor

koff_h 0.019 # Hapten dissociation

lf N*5e-5 # Lyn association with unphoshporylated/phosphorylated beta ITAM via unique/SH2 domain.

lr1 20 # Lyn unique domain dissociation

lr2 0.12 # Lyn SH2 domain dissociation

sf N*6e-5 # Syk association with phosphorylated gamma ITAM

sr 0.20 # Syk dissociation (In Faeder et al.J. Immunol.(2003), sr = 0.13 s-1)

gf N*1.25e-6 # Grb2 association with phosphorylated LAT

gr 0.30 # Grb2 dissociation

plb1_o 30 # FceRI beta phosphorylation mediated by unique domain-bound Lyn in raft regions

plb1_d 6 # FceRI beta phosphorylation mediated by unique domain-bound Lyn in nonraft regions

plb2_o 100 # FceRI beta phosphorylation mediated by SH2 domain-bound Lyn in raft regions

plb2_d 20 # FceRI beta phosphorylation mediated by SH2 domain-bound Lyn in nonraft regions

plg1_o 1 # FceRI gamma phosphorylation mediated by unique domain-bound Lyn in raft regions

plg1_d 0.2 # FceRI gamma phosphorylation mediated by unique domain-bound Lyn in nonraft regions

plg2_o 3 # FceRI gamma phosphorylation mediated by SH2 domain-bound Lyn in raft regions

plg2_d 0.6 # FceRI gamma phosphorylation mediated by SH2 domain-bound Lyn in nonraft regions

pss1 100 # Syk autophosphorylation by receptor-bound Syk not phosphorylated in the activation loop

pss2 200 # Syk autophosphorylation by receptor-bound Syk phosphoryalted in the activation loop

psl N*6e-5 # LAT phosphorylation by receptor-bound Syk

db 20 # FceRI beta dephospohrylation in nonraft regions (in raft regions, the parameter is z*20)

dg 20 # FceRI gamma dephosphorylation in nonraft regions (in raft regions, the parameter is z*20)

ds 20 # Syk dephosphorylation in nonraft regions (in raft regions, the parameter is z*20)

dl 70 # LAT dephosphorylation in nonraft regiosn (in raft regions, the parameter is z*70)

f 0.3 # Lipid raft fraction of the plasma membrane

N 8000 # number of rafts/cell; mean of raft 100 nm; aggregated raft area=raft compartment=0.30% of #cell #membrane,

# cell membrane area=8e-6 cm2 cell;

Tau 10 # Mean raft life time10 s

Phi_r f # Raft partition coefficient for receptor monomer (random distribution)

Phi_d 0.85 # Raft partition coefficient for receptor dimer

Phi_l 0.85 # Raft partition coefficient for Lyn

Phi_t 0.85 # Raft partition coefficient for LAT

r_o (1/Tau)*Phi_r/(1-Phi_r) # r_o=(k_+,R_N) for receptor monomer (see Eq (1) in the paper)

r_d 1/Tau # r_d=Lambda=1/Tau (mean turn over rate of lipid rafts)

rdimer_o (1/Tau)*Phi_d/(1-Phi_d) # rdimer_o=(K_+,D_N) for receptor dimer (see Eq (1) in the paper)

rdimer_d 1/Tau # rdimer_d=Lambda=1/Tau (mean turnover rate of lipid rafts)

l_o (1/Tau)*Phi_l/(1-Phi_l) # l_o=(k_+,L_N) for Lyn (see Eq (1) in the paper)

l_d 1/Tau # l_d=Lambda=1/Tau (mean turnover rate of lipid rafts)

t_o (1/Tau)*Phi_t/(1-Phi_t) # t_o=(k_+,T_N) for LAT (see Eq (1) in the paper)

t_d 1/Tau # t_d=Lambda=1/Tau (mean turnover rate of lipid rafts)

z=0.1 # Lipid raft protection coeff;

# z=(Rate of protein dephosphorylation inside rafts)/(Rate of protein dephosphorylation outside rafts)

**end parameters**

**begin molecule types**

L(l,l)

FCR(s~d~o,a,b~Y~pY,g~Y~pY)

Lyn(s~d~o,U,SH2)

Syk(tSH2,a~Y~pY)

LAT(s~d~o,p~Y~pY)

Grb2(SH2)

#Hap(l)

**end molecule types**

**begin species**

L(l,l) LigT

FCR(s~o,a,b~Y,g~Y) RecT*r_o/(r_o + r_d)

FCR(s~d,a,b~Y,g~Y) RecT*r_d/(r_o + r_d)

Lyn(s~o,U,SH2) LynT*l_o/(l_o + l_d)

Lyn(s~d,U,SH2) LynT*l_d/(l_o + l_d)

Syk(tSH2,a~Y) SykT

LAT(s~o,p~Y) LATT*t_o/(t_o + t_d)

LAT(s~d,p~Y) LATT*t_d/(t_o + t_d)

Grb2(SH2) GrbT

#Hap(l) HapT;

**end species**

**begin reaction rules**

# Ligand binding and receptor crosslinking

FCR(a) + L(l,l) <-> FCR(a!1).L(l!1,l) kon, koff # Binding from solution

FCR(s~d,a) + L(l,l!1).FCR(s~d,a!1) <-> FCR(s~d,a!2).L(l!2,l!1).FCR(s~d,a!1) kx, koff

FCR(s~o,a) + L(l,l!1).FCR(s~o,a!1) <-> FCR(s~o,a!2).L(l!2,l!1).FCR(s~o,a!1) kx, koff

# Hapten binding

#FCR(a) + Hap(l) <-> FCR(a!1).Hap(l!1) kon_h, koff_h

# Receptor - Lyn interaction

FCR(s~d,b~Y) + Lyn(s~d,U,SH2) <-> FCR(s~d,b~Y!1).Lyn(s~d,U!1,SH2) lf, lr1

FCR(s~d,b~pY) + Lyn(s~d,U,SH2) <-> FCR(s~d,b~pY!1).Lyn(s~d,U,SH2!1) lf, lr2

FCR(s~o,b~Y) + Lyn(s~o,U,SH2) <-> FCR(s~o,b~Y!1).Lyn(s~o,U!1,SH2) lf, lr1

FCR(s~o,b~pY) + Lyn(s~o,U,SH2) <-> FCR(s~o,b~pY!1).Lyn(s~o,U,SH2!1) lf, lr2

# Receptor-Syk binding

Syk(tSH2) + FCR(g~pY) <-> Syk(tSH2!1).FCR(g~pY!1) sf, sr

# LAT-Grb2 binding

LAT(p~pY) + Grb2(SH2) <-> LAT(p~pY!1).Grb2(SH2!1) gf, gr

# Receptor phosphorylation by Lyn:

Lyn(s~o,U!1,SH2).FCR(s~o,b~Y!1).FCR(s~o,b~Y) -> Lyn(s~o,U!1,SH2).FCR(s~o,b~Y!1).FCR(s~o,b~pY) plb1_o

Lyn(s~o,U,SH2!1).FCR(s~o,b~pY!1).FCR(s~o,b~Y) -> Lyn(s~o,U,SH2!1).FCR(s~o,b~pY!1).FCR(s~o,b~pY) plb2_o

Lyn(s~o,U!1,SH2).FCR(s~o,b~Y!1).FCR(s~o,g~Y) -> Lyn(s~o,U!1,SH2).FCR(s~o,b~Y!1).FCR(s~o,g~pY) plg1_o

Lyn(s~o,U,SH2!1).FCR(s~o,b~pY!1).FCR(s~o,g~Y) -> Lyn(s~o,U,SH2!1).FCR(s~o,b~pY!1).FCR(s~o,g~pY) plg2_o

Lyn(s~d,U!1,SH2).FCR(s~d,b~Y!1).FCR(s~d,b~Y) -> Lyn(s~d,U!1,SH2).FCR(s~d,b~Y!1).FCR(s~d,b~pY) plb1_d

Lyn(s~d,U,SH2!1).FCR(s~d,b~pY!1).FCR(s~d,b~Y) -> Lyn(s~d,U,SH2!1).FCR(s~d,b~pY!1).FCR(s~d,b~pY) plb2_d

Lyn(s~d,U!1,SH2).FCR(s~d,b~Y!1).FCR(s~d,g~Y) -> Lyn(s~d,U!1,SH2).FCR(s~d,b~Y!1).FCR(s~d,g~pY) plg1_d

Lyn(s~d,U,SH2!1).FCR(s~d,b~pY!1).FCR(s~d,g~Y) -> Lyn(s~d,U,SH2!1).FCR(s~d,b~pY!1).FCR(s~d,g~pY) plg2_d

# Syk autophosphorylation

Syk(a~Y).Syk(a~Y) -> Syk(a~Y).Syk(a~pY) pss1

Syk(a~pY).Syk(a~Y) -> Syk(a~pY).Syk(a~pY) pss2

# LAT phosphorylation by Syk

Syk(tSH2!1).FCR(s~d,g~pY!1) + LAT(s~d,p~Y) -> Syk(tSH2!1).FCR(s~d,g~pY!1) + LAT(s~d,p~pY) psl

Syk(tSH2!1).FCR(s~o,g~pY!1) + LAT(s~o,p~Y) -> Syk(tSH2!1).FCR(s~o,g~pY!1) + LAT(s~o,p~pY) psl

# Receptor dephosphorylation

FCR(s~d,b~pY) -> FCR(s~d,b~Y) db

FCR(s~d,g~pY) -> FCR(s~d,g~Y) dg

FCR(s~o,b~pY) -> FCR(s~o,b~Y) z*db

FCR(s~o,g~pY) -> FCR(s~o,g~Y) z*dg

# Syk dephosphorylation (at membrane)

FCR(s~d,g~pY!1).Syk(tSH2!1,a~pY) -> FCR(s~d,g~pY!1).Syk(tSH2!1,a~Y) ds

FCR(s~o,g~pY!1).Syk(tSH2!1,a~pY) -> FCR(s~o,g~pY!1).Syk(tSH2!1,a~Y) z*ds

# Syk dephosphorylation (at cytosol)

Syk(tSH2,a~pY) -> Syk(tSH2,a~Y) ds

# LAT dephosphorylation

LAT(s~d,p~pY) -> LAT(s~d,p~Y) dl

LAT(s~o,p~pY) -> LAT(s~o,p~Y) z*dl

# Raft - non-raft transition

FCR(s~d,a,b~Y) <-> FCR(s~o,a,b~Y) r_o, r_d

FCR(s~d,a,b~pY) <-> FCR(s~o,a,b~pY) r_o, r_d

FCR(s~d,a!1,b~Y).L(l!1,l) <-> FCR(s~o,a!1,b~Y).L(l!1,l) r_o, r_d

FCR(s~d,a!1,b~pY).L(l!1,l) <-> FCR(s~o,a!1,b~pY).L(l!1,l) r_o, r_d

Lyn(s~d,U,SH2) <-> Lyn(s~o,U,SH2) l_o, l_d

FCR(s~d,a,b~Y!1).Lyn(s~d,U!1) <-> FCR(s~o,a,b~Y!1).Lyn(s~o,U!1) l_o, l_d

FCR(s~d,a,b~pY!1).Lyn(s~d,SH2!1) <-> FCR(s~o,a,b~pY!1).Lyn(s~o,SH2!1) l_o, l_d

FCR(s~d,a!1,b~Y!2).L(l!1,l).Lyn(s~d,U!2) <-> FCR(s~o,a!1,b~Y!2).L(l!1,l).Lyn(s~o,U!2) l_o, l_d

FCR(s~d,a!1,b~pY!2).L(l!1,l).Lyn(s~d,SH2!2) <-> FCR(s~o,a!1,b~pY!2).L(l!1,l).Lyn(s~o,SH2!2) l_o, l_d

FCR(s~d,a!1,b~Y).L(l!1,l!2).FCR(s~d,a!2,b~Y) <-> FCR(s~o,a!1,b~Y).L(l!1,l!2).FCR(s~o,a!2,b~Y) rdimer_o, rdimer_d

FCR(s~d,a!1,b~pY).L(l!1,l!2).FCR(s~d,a!2,b~Y) <-> FCR(s~o,a!1,b~pY).L(l!1,l!2).FCR(s~o,a!2,b~Y) rdimer_o, rdimer_d

FCR(s~d,a!1,b~pY).L(l!1,l!2).FCR(s~d,a!2,b~pY) <-> FCR(s~o,a!1,b~pY).L(l!1,l!2).FCR(s~o,a!2,b~pY) rdimer_o, rdimer_d

FCR(s~d,a!1,b~Y!3).L(l!1,l!2).FCR(s~d,a!2,b~Y).Lyn(s~d,U!3) <->\

FCR(s~o,a!1,b~Y!3).L(l!1,l!2).FCR(s~o,a!2,b~Y).Lyn(s~o,U!3) l_o, l_d

FCR(s~d,a!1,b~Y!3).L(l!1,l!2).FCR(s~d,a!2,b~pY).Lyn(s~d,U!3) <->\

FCR(s~o,a!1,b~Y!3).L(l!1,l!2).FCR(s~o,a!2,b~pY).Lyn(s~o,U!3) l_o, l_d

FCR(s~d,a!1,b~pY!3).L(l!1,l!2).FCR(s~d,a!2,b~Y).Lyn(s~d,SH2!3) <->\

FCR(s~o,a!1,b~pY!3).L(l!1,l!2).FCR(s~o,a!2,b~Y).Lyn(s~o,SH2!3) l_o, l_d

FCR(s~d,a!1,b~pY!3).L(l!1,l!2).FCR(s~d,a!2,b~pY).Lyn(s~d,SH2!3) <->\

FCR(s~o,a!1,b~pY!3).L(l!1,l!2).FCR(s~o,a!2,b~pY).Lyn(s~o,SH2!3) l_o, l_d

FCR(s~d,a!1,b~Y!3).L(l!1,l!2).FCR(s~d,a!2,b~Y!4).Lyn(s~d,U!3).Lyn(s~d,U!4) <->\

FCR(s~o,a!1,b~Y!3).L(l!1,l!2).FCR(s~o,a!2,b~Y!4).Lyn(s~o,U!3).Lyn(s~o,U!4) l_o, l_d

FCR(s~d,a!1,b~pY!3).L(l!1,l!2).FCR(s~d,a!2,b~Y!4).Lyn(s~d,SH2!3).Lyn(s~d,U!4) <->\

FCR(s~o,a!1,b~pY!3).L(l!1,l!2).FCR(s~o,a!2,b~Y!4).Lyn(s~o,SH2!3).Lyn(s~o,U!4) l_o, l_d

FCR(s~d,a!1,b~pY!3).L(l!1,l!2).FCR(s~d,a!2,b~pY!4).Lyn(s~d,SH2!3).Lyn(s~d,SH2!4) <->\

FCR(s~o,a!1,b~pY!3).L(l!1,l!2).FCR(s~o,a!2,b~pY!4).Lyn(s~o,SH2!3).Lyn(s~o,SH2!4) l_o, l_d

LAT(s~d) <-> LAT(s~o) t_o, t_d

**end reaction rule**s

**begin observables**

Molecules pBeta FCR(b~pY!?)

Molecules pGamma FCR(g~pY!?)

Molecules pSyk Syk(tSH2!+,a~pY)

Molecules pLAT LAT(p~pY!?)

**end observables**

**generate_network**({overwrite=>1});

**simulate_ode**({t_end=>3600, n_steps=>3600,atoll=>1e-08,rtol=>1e-08,sparse=>1});
